# Manganese/bipyridine-catalyzed non-directed C(sp^3^)–H bromination using NBS and TMSN_3_

**DOI:** 10.3762/bjoc.17.74

**Published:** 2021-04-22

**Authors:** Kumar Sneh, Takeru Torigoe, Yoichiro Kuninobu

**Affiliations:** 1 Department of Molecular and Material Sciences, Interdisciplinary Graduate School of Engineering Sciences, Kyushu University, 6-1 Kasugakoen, Kasuga-shi, Fukuoka 816-8580, Japan; 2 Institute for Materials Chemistry and Engineering, Kyushu University, 6-1 Kasugakoen, Kasuga-shi, Fukuoka 816-8580, Japan

**Keywords:** bromination, C–H transformation, hydrogen abstraction, manganese, radical

## Abstract

A Mn(II)/bipyridine-catalyzed bromination reaction of unactivated aliphatic C(sp^3^)−H bonds has been developed using *N*-bromosuccinimide (NBS) as the brominating reagent. The reaction proceeded in moderate-to-good yield, even on a gram scale. The introduced bromine atom can be converted into fluorine and allyl groups.

## Introduction

Organic halides are versatile precursors for various synthetic protocols and are frequently used to introduce a variety of functionalities, such as boron-, silicon-, nitrogen-, and oxygen-based functional groups, and in C−C bond forming reactions, such as cross-coupling reactions [1–6]. The traditional method used for the preparation of alkyl bromides is the reaction of their corresponding alkyl alcohols with HBr, PBr_3_, or other brominating reagents [7–13].

**Scheme 1:**
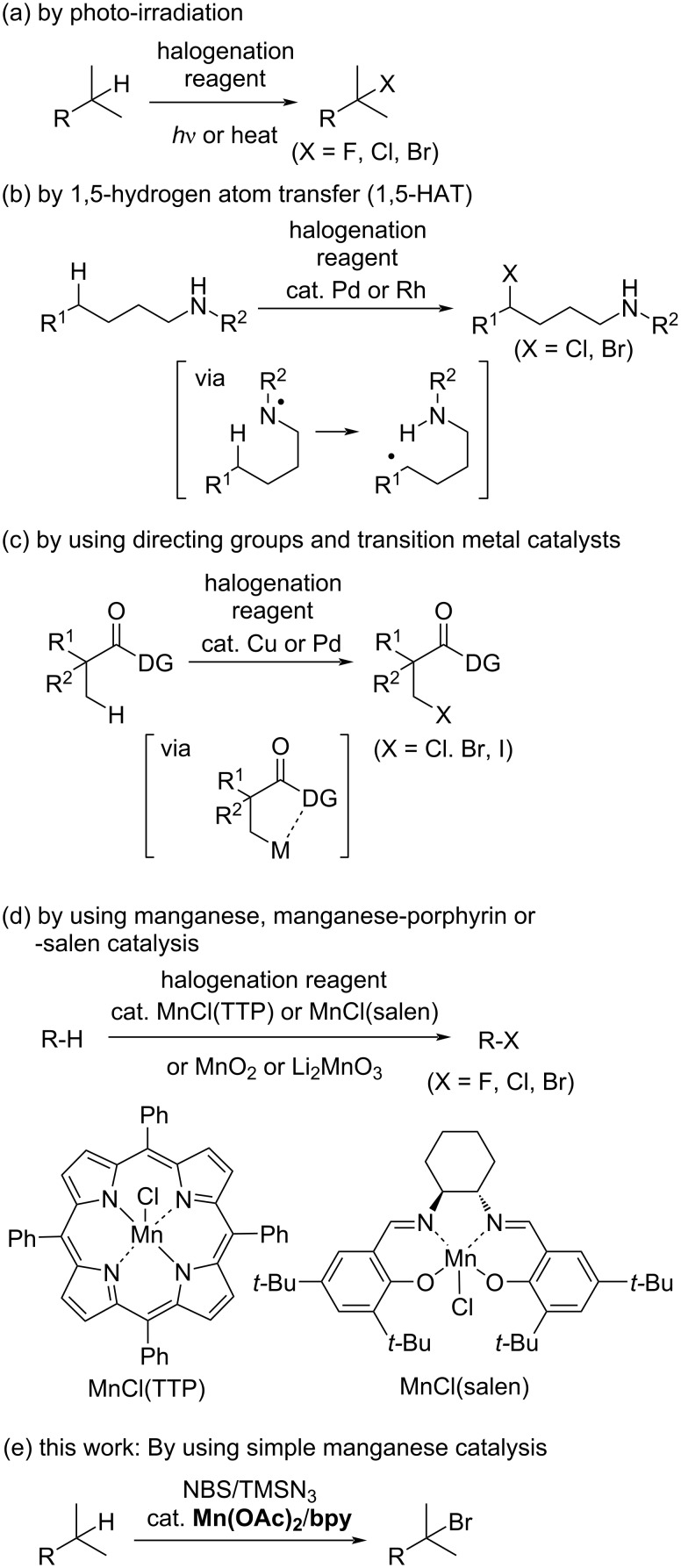
Several examples of C(sp^3^)–H halogenation.

Direct C–H halogenation is one of the most efficient methods used for the synthesis of halogenated organic molecules. This direct method involves the reaction of an alkane with Br_2_, CBr_4_, or H_2_O_2_–HBr under photolysis or at high temperatures in the absence of a catalyst (Scheme 1a) [14–16]. However, these reactions do not exhibit any selectivity due to the indiscriminate attack of bromine radicals on the C–H bonds in the substrate, which leads to the formation of a mixture of halogenated products. Electrophilic and radical C(sp^3^)−H halogenation at the benzylic and allylic position using *N*-halosuccinimide with azobisisobutyronitrile or benzoyl peroxide as a radical initiator is known as the Wohl–Ziegler bromination reaction, which requires heating, acidic/basic conditions, and/or UV irradiation (Scheme 1a) [17–20].

There are several types of transition-metal-catalyzed C(sp^3^)−H halogenation reactions reported in the literature (Scheme 1b–d). Transition-metal-catalyzed 1,5-hydrogen atom transfer (1,5-HAT) is effective for promoting regioselective C(sp^3^)−H halogenation reactions (Scheme 1b) [21–23]. The regioselectivity is controlled by the formation of a six-membered cyclic intermediate. Directing-group-assisted C(sp^3^)−H halogenation reactions are efficient for promoting regioselective C(sp^3^)−H halogenations (Scheme 1c) [24–28]. In these reactions, the C(sp^3^)–H bond at the β-position of an oxazoline or amide is selectively activated using a copper or palladium catalyst.

Manganese is one of the most abundant and nontoxic transition metals found in the earth’s crust and its corresponding complexes and salts are useful in synthetic organic reactions [29–43]. Highly reactive and selective bromination reactions have been achieved using a stoichiometric amount of MnO_2_ [44] or a catalytic amount of Li_2_MnO_3_ [45] under fluorescent light irradiation in the presence of Br_2_ (Scheme 1d). Hill [46] and Groves [47–49] have reported the manganese-porphyrin-catalyzed chlorination and bromination of C(sp^3^)−H bonds, respectively (Scheme 1d). Groves et al. also reported the manganese-salen-catalyzed fluorination of benzylic C(sp^3^)−H bonds [49]. Although these methods are efficient, they have a limited substrate scope (cycloalkanes and substrates bearing a benzylic C–H group). Therefore, there remains room for the development of a simple manganese catalytic system to achieve C(sp^3^)−H halogenation using commercially available reagents.

Herein, we report a manganese-catalyzed C(sp^3^)–H bromination reaction at the methine and benzylic positions of a wide range of substrates. The manganese catalyst, brominating agent, and additives are commercially available, and the reaction can be achieved by simply mixing these reagents with the substrate.

## Results and Discussion

The reaction of isoamyl alcohol derivative **1a** with *N*-bromosuccinimide (NBS) and TMSN_3_ in the presence of a catalytic amount of Mn(OAc)_2_ and bipyridine (bpy) in 1,2-dichloroethane (DCE) at 60 °C for 18 h gave C(sp^3^)–H brominated product **2a** in 10% yield (Table 1, entry 1). Although the yield of **2a** did not increase when performing the reaction in acetonitrile (Table 1, entry 2), the yield of **2a** was dramatically improved to 62% using PhCF_3_ as the solvent (Table 1, entry 3). Other manganese salts, such as MnBr_2_ and Mn(acac)_2_, were also effective in the reaction, giving similar yields (Table 1, entries 4 and 5). Other first-row transition metal salts, such as Fe(OAc)_2_ and Co(OAc)_2_, did not improve the yield of **2a** (Table 1, entries 6 and 7). Product **2a** was formed in 21 and 49% yields, respectively when the reaction was conducted in the absence of the transition metal salt and bpy ligand (Table 1, entries 8 and 9). TMSN_3_ was indispensable in this reaction because the C(sp^3^)–H bromination reaction did not occur in its absence (Table 1, entry 10). We then investigated the following experiments using the conditions described in entry 3.

**Table 1:** Optimization of reaction conditions^a^.

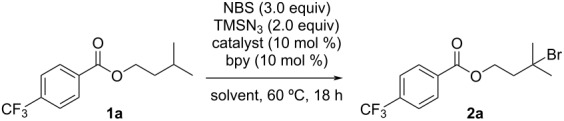			

entry	catalyst	solvent	yield (%)^b^

1	Mn(OAc)_2_	DCE	10
2	Mn(OAc)_2_	MeCN	10
3	Mn(OAc)_2_	PhCF_3_	62 (53)^c^
4	MnBr_2_	PhCF_3_	55
5	Mn(acac)_2_	PhCF_3_	54
6	Fe(OAc)_2_	PhCF_3_	42
7	Co(OAc)_2_	PhCF_3_	30
8	–	PhCF_3_	21
9^d^	Mn(OAc)_2_	PhCF_3_	49
10^e^	Mn(OAc)_2_	PhCF_3_	<1

^a^Conditions: **1a** (0.100 mmol, 1.0 equiv), NBS (0.300 mmol, 3.0 equiv), TMSN_3_ (0.200 mmol, 2.0 equiv), catalyst (10 mol %), bpy (10 mol %), solvent (0.50 mL). ^b^The ^1^H NMR yields were determined using 1,1,2,2-tetrachloroethane as an internal standard. ^c^Isolated yield. ^d^Without bpy. ^e^Without TMSN_3_.

**Scheme 2:**
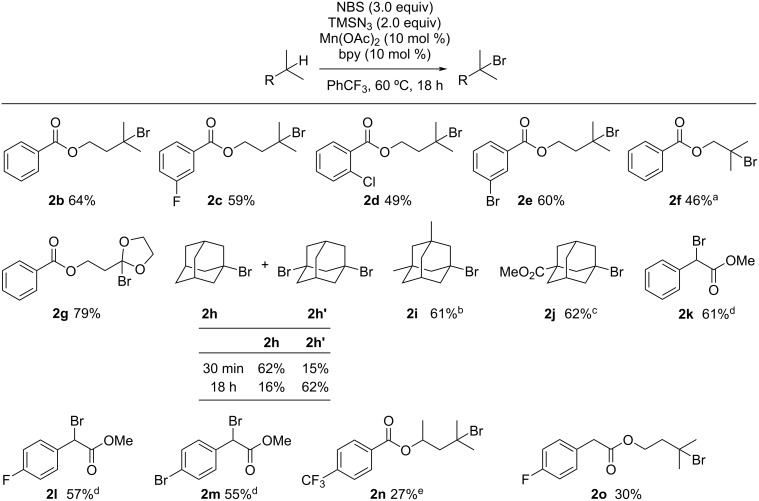
Substrate scope. ^a^80 °C. ^b^45 min. ^c^4 h. ^d^90 °C, ^e^GC yield of mono-brominated product **2n** using mesitylene as internal standard.

Under the optimized reaction conditions, we investigated the C(sp^3^)–H bromination reaction of several substrates (Scheme 2). The reaction proceeded regioselectively at the methine C(sp^3^)–H bond of isoamyl benzoate (**1b**) to give **2b** in 64% yield. Isoamyl benzoates bearing halogen atoms, such as fluorine, chlorine, or bromine, on the phenyl ring were also suitable substrates and gave C(sp^3^)–H brominated products **2c**–**e** in 49–60% yields, without any loss of the halogen substituents. Although the C(sp^3^)–H bromination of isobutyl benzoate **1f** did not proceed at 60 °C, the corresponding C(sp^3^)–H brominated compound **2f** was produced at higher temperature (80 °C). The C(sp^3^)–H bond in acetal **1g** was efficiently brominated to give **2g** in 79% yield. The reaction of adamantane (**1h**) proceeded selectively at the tertiary C(sp^3^)–H bond to give a mixture of mono- and dibrominated products (**2h** and **2h′**). The selectivity of **2h** and **2h′** can be controlled by varying the reaction time; mono-brominated **2h** was obtained in 62% yield as the major product after 30 min of reaction and dibrominated **2h′** was afforded as the major product after 18 h. Similarly, 1,3-dimethyladamantane (**1i**) and methyl adamantane-1-carboxylate (**1j**) were successfully converted to brominated products **2i** and **2j**, respectively. For benzeneacetic acid methyl esters **1k**, **1l** and **1m**, the C(sp^3^)–H bromination reaction proceeded selectively at the benzylic position and their corresponding brominated products (**2k**, **2l** and **2m**) were obtained in 61, 57 and 55% yield, respectively.

We next investigated the regioselectivity of the reaction using substrates with two possible reaction sites. The reaction of substrate **1n** bearing two methine C(sp^3^)–H bonds occurred selectively at the terminal position giving product **2n** in 27% yield. The C(sp^3^)–H bromination reaction took place selectively at the methine C(sp^3^)–H bond when using substrate **1o**, which has both methine and benzylic C(sp^3^)–H bonds, which gave product **2o** in 30% yield.

**Scheme 3:**
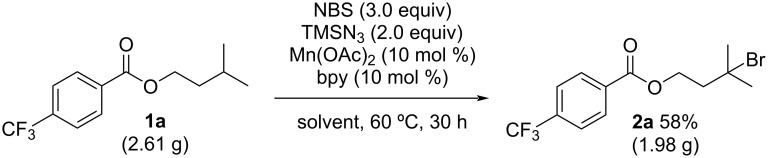
Gram-scale synthesis of **2a**.

The manganese-catalyzed C(sp^3^)–H bromination reaction proceeded in good yield, even on a gram scale. The reaction was performed using 2.61 g of **1a** with NBS and TMSN_3_ in the presence of a catalytic amount of Mn(OAc)_2_ and bpy to give 1.98 g of **2a** in 58% yield (Scheme 3).

**Scheme 4:**
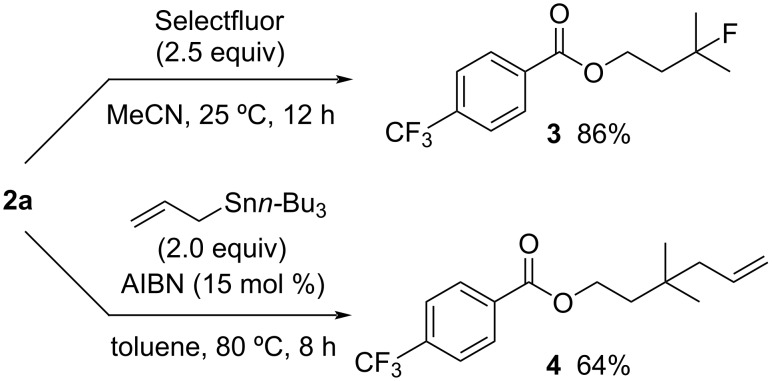
Conversion of the C(sp^3^)–Br bond.

The introduced bromine atom can be converted into other functional groups. The reaction of **2a** with selectfluor in MeCN at 25 °C for 12 h gave fluorinated product **3** in 86% yield (Scheme 4, top) [50]. Allylated product **4** was obtained in 64% yield upon treating **2a** with allyltributylstannane in the presence of a catalytic amount of AIBN (Scheme 4, bottom) [51].

**Scheme 5:**
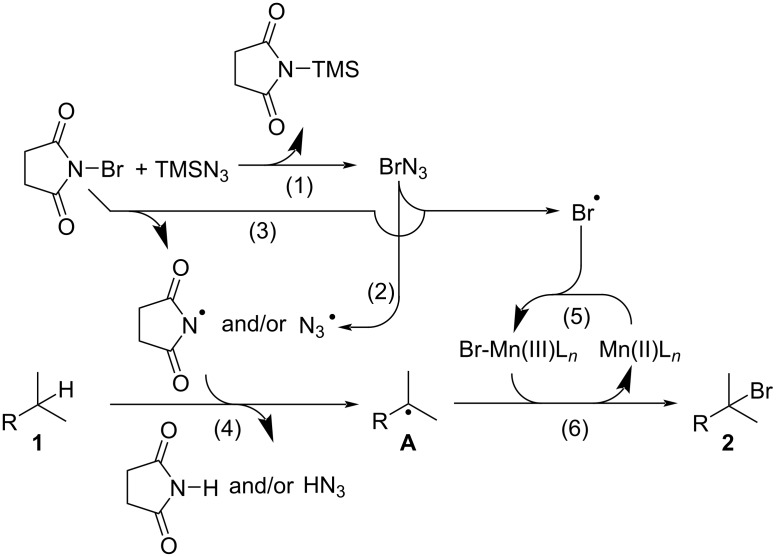
Proposed mechanism of manganese-catalyzed C(sp^3^)–H bromination.

Table 1 shows that the C(sp^3^)–H bromination reaction proceeds in the absence of a transition metal salt or bpy ligand, and was accelerated by transition metal salts, especially Mn(OAc)_2_. In addition, the results also suggest that TMSN_3_ is required for the C(sp^3^)–H bromination reaction. The proposed reaction mechanism is shown in Scheme 5, which involves the following steps. (1) The reaction between NBS and TMSN_3_ generates bromine azide via the elimination of *N*-(trimethylsilyl)succinimide [52–53]; (2) bromine and azide radicals are then formed via homolytic cleavage of the weak Br–N_3_ bond in bromine azide [54–55]; (3) the bromine radical can also be generated from NBS with the formation of a succinimide radical; (4) alkyl radical intermediate **A** is then formed via hydrogen abstraction by the succinimidyl radical and/or azidyl radical [56–57]; (5) the Br–Mn(III) species is then formed from the Mn(II) catalyst and bromine radical; and (6) brominated product **2** formed by the reaction of intermediate **A** with the Br–Mn(III) species with the regeneration of the Mn(II) catalyst.

## Conclusion

In summary, we have successfully developed a manganese-catalyzed bromination of unactivated aliphatic C(sp^3^)−H bonds. The reaction proceeded selectively at the methine and benzylic positions using simple and commercially available compounds, such as NBS, TMSN_3_, Mn(OAc)_2_, and bpy, even on a gram scale. Furthermore, the brominated products can be easily functionalized upon the introduction of other functional groups, such as fluorine and allyl groups. We hope that this C(sp^3^)–H bromination reaction will become a useful method to synthesize organic compounds with bromine atom(s).

## Supporting Information

File 1 Experimental procedures, compound characterization data, and copies of ^1^H and ^13^C NMR spectra.
