# Supplementary material for: Manganese/bipyridine-catalyzed non-directed C(sp3)–H bromination using NBS and TMSN3
Source: Beilstein J Org Chem. 2021 Apr 22;17:885–90. doi: 10.3762/bjoc.17.74 (PMC8077618; doi:10.3762/bjoc.17.74)

BEILSTEIN JOURNAL OF ORGANIC CHEMISTRY

Supporting Information

for

Manganese/bipyridine-catalyzed non-directed C(sp^3)–H bromination using NBS and $TMSN_3$

Kumar Sneh, Takeru Torigoe and Yoichiro Kuninobu

Beilstein J. Org. Chem. **2021,** *17,* 885–890. doi:10.3762/bjoc.17.74

Experimental procedures, compound characterization data, and copies of ^{1}H and ^{13}C NMR spectra

License and Terms: This is a supporting information file under the terms of the Creative Commons Attribution License (https://creativecommons.org/licenses/by/4.0). Please note that the reuse, redistribution and reproduction in particular requires that the author(s) and source are credited and that individual graphics may be subject to special legal provisions.
The license is subject to the *Beilstein Journal of Organic Chemistry* terms and conditions: (https://www.beilstein-journals.org/bjoc/terms)

Table of Contents

1. General information S2
2. Structures of substrates S2
3. Experimental section S3
   3-1. Preparation of substrates S3
   3-2. Screening of reaction conditions S6
   3-3. C(sp^3)–H bromination S8
   3-4. Conversion of introduced bromine atom S14
4. References S16
5. ^{1}H and ^{13}C NMR spectra S17

1. General information

All reactions were carried out under nitrogen atmosphere unless otherwise noted. Toluene (anhydrous, Wako), dichloromethane (anhydrous, Wako), THF (anhydrous, Wako), ethyl acetate (anhydrous, Wako), and DMF (anhydrous, Wako) were used as received from commercial sources. MeCN and $PhCF_3$ was distilled over CaH_2 prior to use. Other reagents were purchased from commercial sources and used without further purification. ^{1}H (400 MHz), ^{13}C (100 MHz), and ^{19}F (368 MHz) NMR spectra were recorded using a JEOL ECZ400 spectrometer. High resolution mass spectra were recorded on JEOL JMS-700 (EI) spectrometer.

2. Structures of substrates

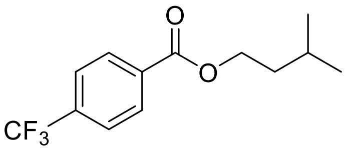
1a

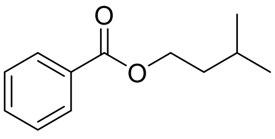
1b

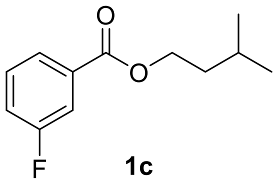
1c

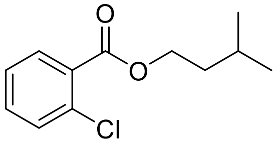
1d

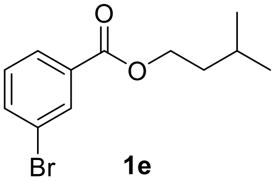
1e

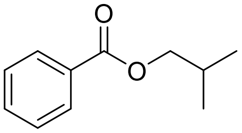
1f

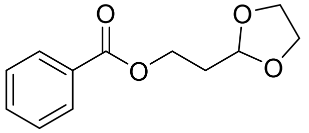
1g

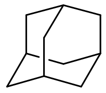
1h

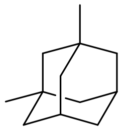
1i

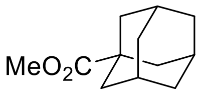
1j

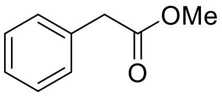
1k

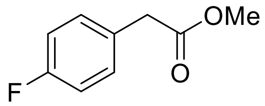
1l

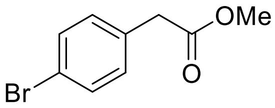
1m

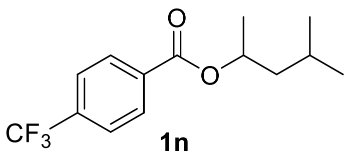
1n

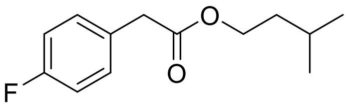
1o

3. Experimental section

3-1. Preparation of substrates

Substrates **1h**, **1i**, and **1k** are commercially available. Substrates **1a–g**, **1j**, **1l–o** were prepared by following the literature procedure.[1] Substrates **1a**, **1c**, **1e**, **1n**, and **1o** are unknown compounds, and other substrates are known compounds. All other starting materials, solvents, and reagents were purchased and used as received.

General procedure 1:

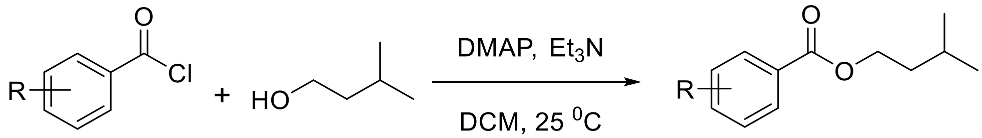

To a solution of alcohol (5.00 mmol, 1.0 equiv), DMAP (4-dimethylaminopyridine, 1.00 mmol, 0.20 equiv) and Et_3N (1.00 mL, 7.50 mmol, 1.5 equiv) in DCM (25.0 mL) at 0 °C was added benzoyl chloride derivative (6.00 mmol, 1.2 equiv). The mixture was warmed to 25 °C and stirred for 6 h. The reaction mixture was quenched with H_2O (5.0 mL) and extracted with DCM (3 × 10 mL). The combined organic layer was dried over $MgSO_4$. The filtrate was concentrated in vacuo and the residue was purified by chromatography on silica gel (hexanes/EtOAc) to afford the desired benzoate.

General procedure 2:

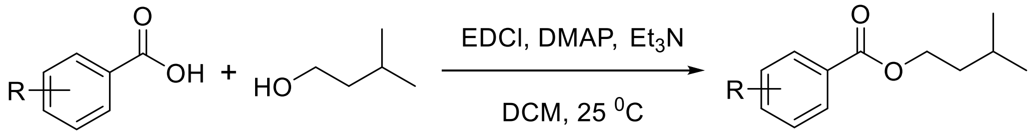

To a solution of benzoic acid derivative (7.00 mmol, 1.0 equiv), DMAP (4-dimethylaminopyridine) (1.40 mmol, 0.20 equiv) and Et_3N (14.0 mmol, 2.0 equiv) in DCM (50.0 mL) at 25 °C were added EDCI (1-ethyl-(3-(3-dimethylamino)propyl)carbodiimide hydrochloride) (14.0 mmol, 2.00 equiv) and alcohol (7.00 mmol, 1.0 equiv). The mixture was

warmed to 25 °C and stirred for 6 h. The reaction mixture was quenched with H_2O (10 mL) and extracted with DCM (3 × 20 mL). The combined organic layer was dried over $MgSO_4$. The filtrate was concentrated in vacuo and the residue was purified by chromatography on silica gel (hexanes/EtOAc) to afford the desired benzoate.

Isopentyl 4-(trifluoromethyl)benzoate (1a)

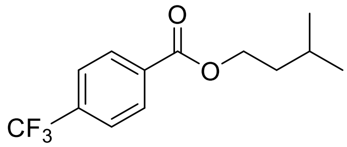

Substrate **1a** was synthesized according to general procedure 2; purified by column chromatography on silica gel (hexane/ethyl acetate = 50:1); colorless liquid (79% yield): ^{1}H NMR (400 MHz, $CDCl_3$) δ 8.14 (d, *J* = 8.0 Hz, 2H), 7.69 (d, *J* = 8.0 Hz, 2H), 4.39 (t, *J* = 6.6 Hz, 2H), 1.84-1.74 (m, 1H), 1.68 (td, *J* = 6.6, 6.6 Hz, 2H), 0.98 (d, *J* = 6.4 Hz, 6H); ^{13}C NMR (100 MHz, $CDCl_3$) δ 165.4, 134.3 (q, *J* = 32.6 Hz), 133.7, 129.9, 125.3 (q, *J* = 3.8 Hz), 123.6 (q, *J* = 271 Hz), 64.2, 37.3, 25.2, 22.5; ^{19}F NMR (376 MHz, $CDCl_3$) δ -63.0; HRMS (ESI^+) *m*/*z*: [M^+] Calcd. For $C_{13}H_{16}F_3O_2$: 261.1102; Found 261.1101.

Isopentyl 3-fluorobenzoate (1c)

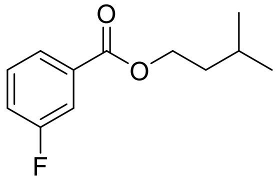

Substrate **1c** was synthesized according to general procedure 1; purified by column chromatography on silica gel (hexane/ethyl acetate = 20:1); colorless liquid (75% yield): ^{1}H NMR (400 MHz, $CDCl_3$) δ 7.81 (ddd, *J* = 8.0, 1.6, 1.6 Hz, 1H), 7.69 (ddd, *J* = 9.2, 1.6, 1.6 Hz, 1H), 7.40 (ddd, *J* = 8.0, 8.0, 5.6 Hz, 1H), 7.23 (ddd, *J* = 8.0, 8.0, 3.2 Hz, 1H), 4.35 (t, *J* = 6.8 Hz, 2H), 1.83-1.73 (m, 1H), 1.65 (td, *J* = 6.8, 6.8 Hz, 2H), 0.96 (d, *J* = 6.4 Hz, 6H); ^{13}C NMR (100 MHz, $CDCl_3$) δ 165.5, 162.5 (d, *J* = 245 Hz), 132.6 (d, *J* = 7.7 Hz), 130.0 (d, *J* = 7.6 Hz), 125.2 (d, *J* = 2.9 Hz), 120.1 (d, *J* = 21.0 Hz), 116.4 (d, *J* = 23.0 Hz), 64.0, 37.3, 25.2, 22.5; ^{19}F NMR (376 MHz, $CDCl_3$) δ -112.4; HRMS (ESI^+) *m*/*z*: [M^+] Calcd. For $C_{12}H_{16}FO_2$: 211.1134; Found 211.1134.

Isopentyl 3-bromobenzoate (1e)

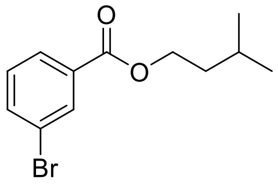

Substrate **1e** was synthesized according to general procedure 2; purified by column chromatography on silica gel (hexane/ethyl acetate = 20:1); colorless liquid (80% yield): ^{1}H NMR (400 MHz, $CDCl_3$) δ 8.16 (dd, *J* = 1.6, 1.6 Hz, 1H), 7.96 (ddd, *J* = 8.4, 1.2, 1.2 Hz, 1H), 7.69-7.65 (m, 1H), 7.31 (dd, *J* = 7.8, 7.8 Hz, 1H), 4.36 (t, *J* = 6.6 Hz, 2H), 1.83-1.73 (m, 1H), 1.66 (td, *J* = 6.9, 6.6 Hz, 2H), 0.97 (d, *J* = 6.4 Hz, 6H); ^{13}C NMR (100 MHz, $CDCl_3$) δ 165.3, 135.7, 132.5, 132.4, 129.9, 128.1, 122.4, 64.1, 37.3, 25.2, 22.5; HRMS (ESI^+) *m/z*: [M^+] Calcd. For $C_{12}H_{15}BrO_2$, 270.0255; Found 270.0255.

4-Methylpentan-2-yl 4-(trifluoromethyl)benzoate (1n)

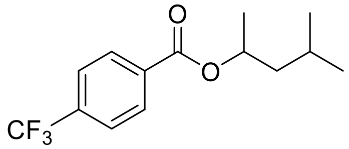

Substrate **1o** was synthesized according to general procedure 2; purified by column chromatography on silica gel (hexane/ethyl acetate = 50:1); colorless liquid (84% yield): ^{1}H NMR (400 MHz, $CDCl_3$) δ 8.15 (d, *J* = 8.0 Hz, 2H), 7.69 (d, *J* = 8.0 Hz, 2H), 5.31-5.23 (m, 1H), 1.77-1.67 (m, 2H), 1.46-1.38 (m, 1H), 1.35 (d, *J* = 5.9 Hz, 3H), 0.94 (d, *J* = 6.4 Hz, 3H), 0.93 (d, *J* = 6.4 Hz, 3H); ^{13}C NMR (100 MHz, $CDCl_3$) δ 165.0, 134.2 (q, *J* = 30.7 Hz), 134.1, 129.9, 125.3 (q, *J* = 3.8 Hz), 123.7 (q, *J* = 271 Hz), 71.0, 45.1, 24.8, 22.9, 22.3, 20.5; ^{19}F NMR (376 MHz, $CDCl_3$) δ -63.0; HRMS (ESI^+) *m/z*: [M^+] Calcd. For $C_{14}H_{18}F_3O_2$: 275.1259; Found 275.1260.

Isopentyl 2-(4-fluorophenyl)acetate (1o)

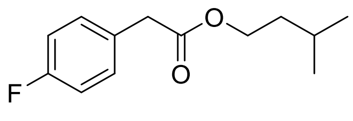

Substrate **1p** was synthesized according to general procedure 2; purified by column chromatography on silica gel (hexane/ethyl acetate = 10:1); colorless liquid (91% yield): ^{1}H NMR (400 MHz, $CDCl_3$) δ 7.23 (dd, *J* = 8.8, 5.5 Hz, 2H), 6.99 (dd, *J* = 8.8, 8.8 Hz, 2H), 4.10 (t, *J* = 6.9 Hz, 2H), 3.57 (s, 2H), 1.68-1.58 (m, 1H), 1.49 (td, *J* = 6.9, 6.9 Hz, 2H), 0.88 (d, *J* = 6.9 Hz, 6H);

^{13}C NMR (100 MHz, $CDCl_3$) δ 171.5, 161.9 (d, J = 244 Hz), 130.8 (d, J = 7.6 Hz), 129.8 (d, J = 3.8 Hz), 115.3 (d, J = 21.0 Hz), 63.6, 40.5, 37.2, 25.0, 22.4; ^{19}F NMR (376 MHz, $CDCl_3$) δ -115.8; HRMS (ESI^+) m/z: [M^+] Calcd. For $C_{13}H_{18}FO_2$: 225.1291; Found 225.1291.

3-2. Screening of reaction conditions

Table S1. Screening of several ligands[a]

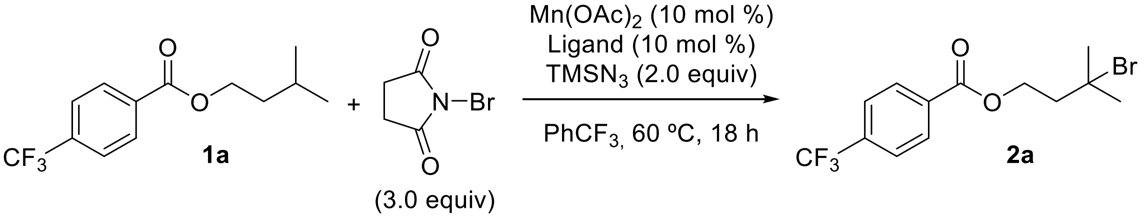

Entry	Ligand	Yield (%)[b]
1	bpy	62
2[c]	bpy	32
3	dtbpy	50
4	phen	65
5	neocuproine	51
6	Me_4phen	61
7	terpyridine	59

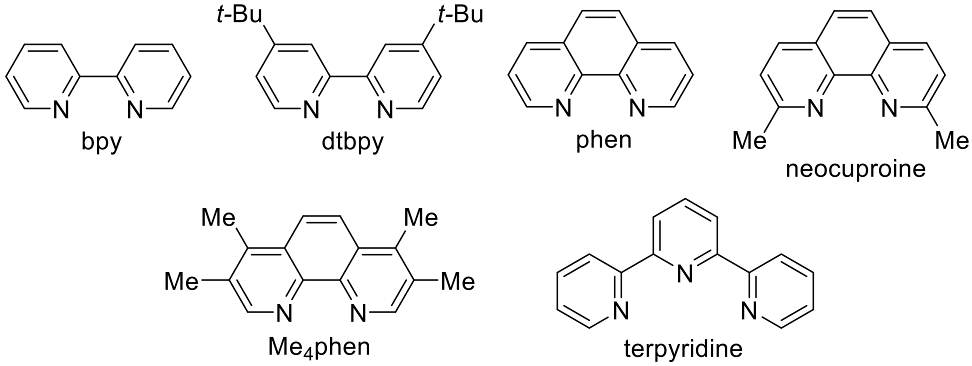

[a]Conditions: **1a** (0.100 mmol, 1.0 equiv), NBS (0.300 mmol, 3.0 equiv), $Mn(OAc)_2$ (10 mol %), ligand (10 mol %), $TMSN_3$ (0.200 mmol, 2.0 equiv), $PhCF_3$ (0.50 mL). [b]^1H NMR yield was determined using 1,1,2,2-tetrachloroethane as an internal standard. [c]Mixed solvent ($PhCF_3$/acetone = 4:1).

The yields obtained by bpy (Entry 1) and phen (Entry 4) were almost the same. Because bpy is cheaper than phen, we utilized bpy as the ligand.

Table S2. Screening of metal salts[a]

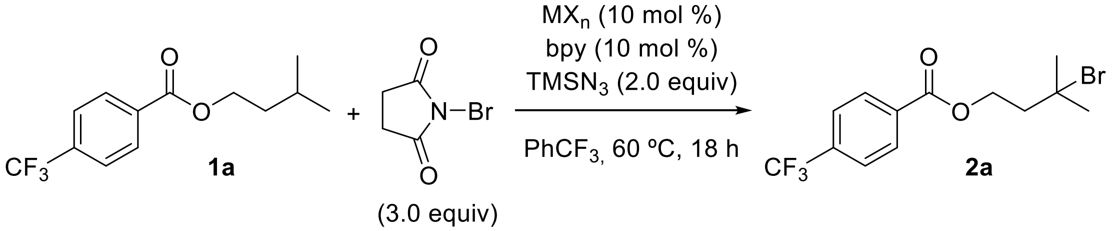

Entry	MX_n	Yield (%)[b]
1	$Mn(TFA)_2$	41
2	$(MeCp)Mn(CO)_3$	43
3	MnF_3	53
4	MnF_3/phen	54
5	$Mn(acac)_2$	54
6	$MnBr_2$	55
7	$MnCl_2$	41
8	$Mn(OAc)_3$	58
9	$Mn(acac)_3$	45

[a]Conditions: **1a** (0.100 mmol, 1.0 equiv), NBS (0.300 mmol, 3.0 equiv), metal salt (10 mol %), bpy (10 mol %), $TMSN_3$ (0.200 mmol, 2.0 equiv), $PhCF_3$ (0.50 mL). [b]^{1}H NMR yield was determined using 1,1,2,2-tetrachloroethane as an internal standard.

Table S3. Screening of halogen sources[a]

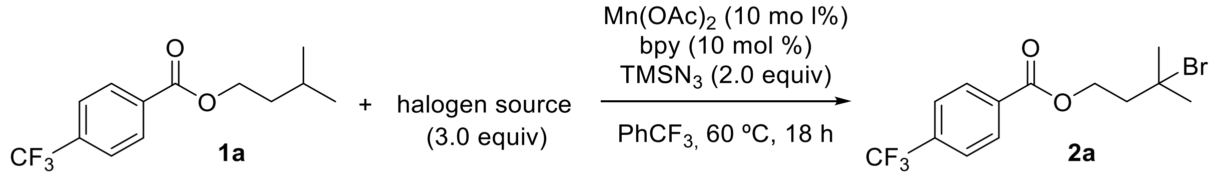

Entry	Halogen Source	Yield (%)[b]
1	NBS	62
2	**A**	42
3	**B**	<1
4	**C**	<1
5	**D**	<1

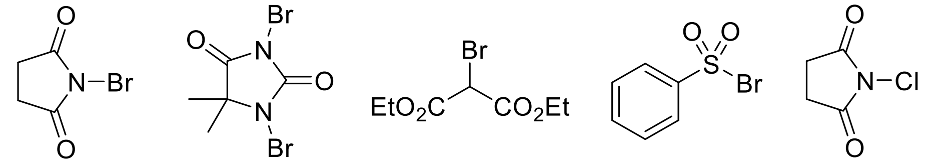

NBS **A** **B** **C** **D**

[a]Conditions: **1a** (0.100 mmol, 1.0 equiv), brominating agent (0.300 mmol, 3.0 equiv), $Mn(OAc)_2$ (10 mol %), bpy (10 mol %), $TMSN_3$ (0.200 mmol, 2.0 equiv), $PhCF_3$ (0.50 mL). [b]^{1}H NMR yield was determined using 1,1,2,2-tetrachloroethane as an internal standard.

3-3. C(sp^3)–H Bromination:

General procedures for C(sp^3)–H bromination

Substrate **1** (0.200 mmol), NBS (0.600 mmol, 31.2 mg), $Mn(OAc)_2$ (0.010 mmol, 2.3 mg), ligand (0.010 mmol, 2.7 mg) were added into a reaction vial (10 mL) with a magnetic stir bar under N_2 atmosphere. $PhCF_3$ (1.0 mL) was added and stirred for 5 min. Then, $TMSN_3$ was added and the vial was sealed with a cap. The reaction mixture was stirred at 60 °C for 18 h. The reaction mixture was cooled to room temperature and diluted with EtOAc. Then the mixture was filtered through a short celite pad and purified by column chromatography on silica gel using hexane/EtOAc mixtures as the eluent.

3-Bromo-3-methylbutyl 4-(trifluoromethyl)benzoate (2a)

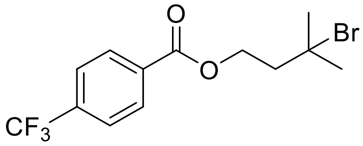

Following the general procedure for the C(sp^3)–H bromination; purified by column chromatography on silica gel (hexane/ethyl acetate = 20:1); colorless liquid (53% yield): ^{1}H NMR (400 MHz, $CDCl_3$) δ 8.15 (d, *J* = 8.2 Hz, 2H), 7.71 (d, *J* = 8.2 Hz, 2H), 4.62 (t, *J* = 6.8 Hz, 2H), 2.32 (t, *J* = 6.8 Hz, 2H), 1.86 (s, 6H); ^{13}C NMR (100 MHz, $CDCl_3$) δ 165.2, 134.5 (q, *J* = 32.6 Hz), 133.3, 130.0, 125.4 (q, *J* = 2.9 Hz), 123.6 (q, *J* = 272 Hz), 63.7, 63.7, 45.3, 34.7; ^{19}F NMR (376 MHz, $CDCl_3$) δ -63.0; HRMS (ESI$^+$) *m*/*z*: [M$^+$] Calcd. for $C_{13}H_{15}BrF_3O_2$, 339.0208; Found 339.0207.

3-Bromo-3-methylbutyl benzoate (2b)[2]

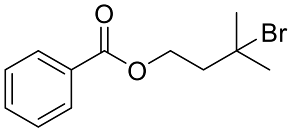

Following the general procedure for the C(sp^3)–H bromination; purified by column chromatography on silica gel (hexane/ethyl acetate = 20:1); colorless liquid (64% yield): ^{1}H NMR (400 MHz, $CDCl_3$) δ 8.04 (dd, *J* = 6.6, 1.6 Hz, 2H), 7.56 (tt, *J* = 7.3, 1.5 Hz, 1H), 7.46-7.42 (m, 2H), 4.58 (t, *J* = 6.6 Hz, 2H), 2.31 (t, *J* = 6.6 Hz, 2H), 1.86 (s, 6H); ^{13}C NMR (100 MHz, $CDCl_3$) δ 166.4, 133.0, 130.1, 129.5, 128.3, 64.2, 63.0, 45.4, 34.7; HRMS (ESI$^+$) *m*/*z*: [M$^+$] Calcd. for $C_{12}H_{16}BrO_2$, 271.0334; Found 271.0334.

3-Bromo-3-methylbutyl 3-fluorobenzoate (2c)

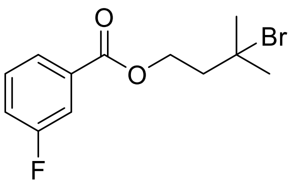

Following the general procedure for the C(sp^3)–H bromination; purified by column chromatography on silica gel (hexane/ethyl acetate = 20:1); colorless liquid (59% yield): ^{1}H NMR (400 MHz, $CDCl_3$) δ 7.83 (ddd, *J* = 7.8, 1.4, 1.4 Hz, 1H), 7.70 (ddd, *J* = 9.3, 1.4, 1.4 Hz, 1H), 7.46-7.38 (m, 1H), 7.27 (ddd, *J* = 8.4, 8.4, 2.4 Hz, 1H), 4.59 (t, *J* = 6.8 Hz, 2H), 2.30 (t, *J* = 6.8

Hz, 2H), 1.86 (s, 6H); ^{13}C NMR (100 MHz, $CDCl_3$) δ 165.3, 162.5 (d, *J* = 245 Hz) , 132.2 (d, *J* = 7.7 Hz), 130.0 (d, *J* = 7.6 Hz), 125.3 (d, *J* = 2.9 Hz), 120.1 (d, *J* = 21.1 Hz, 116.4 (d, *J* = 23.0 Hz), 64.0, 63.5, 45.3, 34.7; ^{19}F NMR (376 MHz, $CDCl_3$) δ -112.2; HRMS (ESI$^+$) *m*/*z*: [M$^+$] Calcd. for $C_{12}H_{15}BrFO_2$, 289.0239; Found 289.0240.

3-Bromo-3-methylbutyl 2-chlorobenzoate (2d)

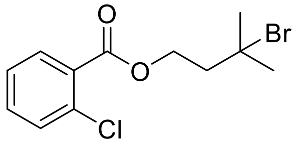

Following the general procedure for the C(sp^3)–H Bromination; purified by column chromatography on silica gel (hexane/ethyl acetate = 20:1); colorless liquid (49% yield): ^{1}H NMR (400 MHz, $CDCl_3$) δ 7.82 (dd, *J* = 7.8, 1.8 Hz, 1H), 7.48-7.39 (m, 2H), 7.34-7.32 (m, 1H), 4.60 (t, *J* = 6.9 Hz, 2H), 2.31 (t, *J* = 6.9 Hz, 2H), 1.85 (s, 6H); ^{13}C NMR (100 MHz, $CDCl_3$) δ 165.6, 133.7, 132.6, 131.3, 131.1, 129.9, 126.6, 64.0, 63.7, 45.2, 34.7; HRMS (ESI$^+$) m/z: [M$^+$] Calcd. for $C_{12}H_{15}$BrClO2, 304.9944; Found 304.9943.

3-Bromo-3-methylbutyl 3-bromobenzoate (2e)

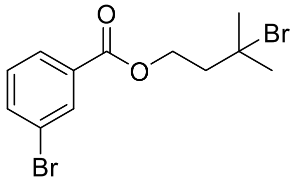

Following the general procedure for the C(sp^3)–H Bromination; purified by column chromatography on silica gel (hexane/ethyl acetate = 20:1); colorless liquid (60% yield): ^{1}H NMR (400 MHz, $CDCl_3$) δ 8.16 (dd, *J* = 1.8, 1.8 Hz, 1H), 7.96 (ddd, *J* = 7.6, 1.2, 1.2 Hz, 1H), 7.71-7.66 (m, 1H), 7.32 (dd, *J* = 7.8, 7.8 Hz, 1H), 4.59 (t, *J* = 6.9 Hz, 2H), 2.30 (t, *J* = 6.9 Hz, 2H), 1.85 (s, 6H); ^{13}C NMR (100 MHz, $CDCl_3$) δ 165.1, 136.0, 132.6, 132.0, 130.0, 128.1, 122.5, 63.9, 63.5, 45.3, 34.7; HRMS (ESI$^+$) *m*/*z*: [M$^+$] Calcd. for $C_{12}H_{15}Br_2O_2$, 348.9439; Found 348.9439.

2-Bromo-2-methylpropyl benzoate (2f)

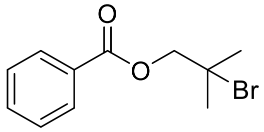

Following the general procedure for the C(sp^3)–H bromination; purified by column chromatography on silica gel (hexane/ethyl acetate = 20:1); colorless liquid (46% yield): ^{1}H NMR (400 MHz, $CDCl_3$) δ 8.09 (dd, *J* = 8.2, 1.6 Hz, 2H), 7.59 (tt, *J* = 7.3, 1.6 Hz, 1H), 7.49-7.45 (m, 2H), 4.45 (s, 2H), 1.86 (s, 6H); ^{13}C NMR (100 MHz, $CDCl_3$) δ 165.9, 133.3, 129.7 (3C), 128.5, 73.3, 60.9, 31.0; HRMS (ESI$^+$) *m*/*z*: [M$^+$] Calcd. for $C_{11}H_{13}BrO_2$, 256.0099; Found 256.0100.

2-(2-Bromo-1,3-dioxolan-2-yl)ethyl benzoate (2g)

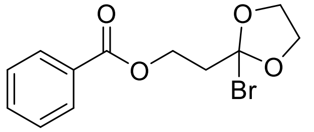

Following the general procedure for the C(sp^3)–H bromination; purified by column chromatography on silica gel (hexane/ethyl acetate = 20:1); colorless liquid (79% yield): ^{1}H NMR (400 MHz, $CDCl_3$) δ 8.02 (dd, *J* = 8.2, 1.6 Hz, 2H), 7.55 (tt, *J* = 7.3, 1.6 Hz, 1H), 7.45-7.41 (m, 2H), 4.61 (t, *J* = 6.4 Hz, 2H), 4.44 (t, *J* = 6.1 Hz, 2H), 3.50 (t, *J* = 6.1 Hz, 2H), 2.84 (t, *J* = 6.4 Hz, 2H); ^{13}C NMR (100 MHz, $CDCl_3$) δ 170.2, 166.2, 133.1, 129.8, 129.6, 128.3, 64.0, 60.1, 33.9, 28.4; HRMS (ESI$^+$) *m*/*z*: [M$^+$] Calcd. for $C_{12}H_{14}BrO_4$, 301.0075; Found 301.0076.

1-Bromoadamantane (2h)[3]

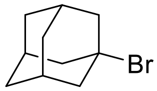

Following the general procedure for the C(sp^3)–H bromination; purified by column chromatography on silica gel (hexane); white solid (62% yield): ^{1}H NMR (400 MHz, $CDCl_3$) δ 2.37 (d, *J* = 3.2 Hz, 6H), 2.14-2.06 (m, 3H), 1.73 (t, *J* = 3.0 Hz, 6H); ^{13}C NMR (100 MHz, $CDCl_3$) δ 66.8, 49.3, 35.5, 32.6; HRMS (ESI$^+$) *m*/*z*: [M$^+$] Calcd. for $C_{10}H_{14}Br$ 213.0279; Found 213.0278.

(1s,3s,5s,7s)-1,3-Dibromoadamantane (2h′)[4]

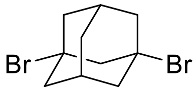

Following the general procedure for the C(sp^3)–H Bromination with reaction time of only 45 min; purified by column chromatography on silica gel (hexane); white solid (62% yield): ^{1}H NMR (400 MHz, $CDCl_3$) δ 2.87 (s, 2H), 2.34-2.25 (m, 10H), 1.70 (t, *J* = 2.5 Hz, 2H); ^{13}C NMR (100 MHz, $CDCl_3$) δ 62.1, 59.0, 56.8, 47.0, 44.9, 35.0, 33.5.

1-Bromo-3,5-dimethyladamantane (2i)[4]

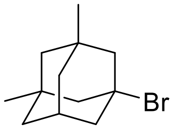

Following the general procedure for the C(sp^3)–H Bromination; purified by column chromatography on silica gel (hexane); white solid (61% yield): ^{1}H NMR (400 MHz, $CDCl_3$) δ 2.20-2.11 (m, 3H), 2.03 (d, *J* = 12.0 Hz, 2H), 1.96 (d, *J* = 12.0 Hz, 2H), 1.41 (d, *J* = 12.4 Hz, 2H), 1.34 (d, *J* = 12.4 Hz, 2H), 1.20 (s, 2H), 0.86 (s, 6H); ^{13}C NMR (100 MHz, $CDCl_3$) δ 66.6, 55.2, 49.7, 47.6, 41.8, 35.5, 32.8, 29.8; HRMS (ESI) *m*/*z*: [M^+] Calcd. for $C_{12}H_{18}Br$, 241.0592; Found 241.0591.

Methyl (1r,3s,5R,7S)-3-bromoadamantane-1-carboxylate (2j)[6]

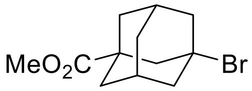

Following the general procedure for the C(sp^3)–H Bromination; purified by column chromatography on silica gel (hexane/ethyl acetate = 20:1); white solid (62% yield): ^{1}H NMR (400 MHz, $CDCl_3$) δ 3.66 (s, 3H), 2.47 (s, 2H), 2.34-2.26 (m, 4H), 2.20 (t, *J* = 2.7 Hz, 2H), 1.90-1.86 (m, 4H), 1.69-1.65 (m, 2H); ^{13}C NMR (100 MHz, $CDCl_3$) δ 175.9, 63.7, 51.9, 49.6, 48.1, 44.9, 37.1, 34.4, 31.7.

Methyl 2-bromo-2-phenylacetate (2k)[7]

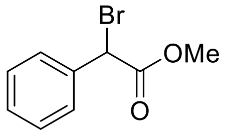

Following the general procedure for the C(sp^3)–H Bromination; purified by column chromatography on silica gel (hexane/ethyl acetate = 20:1); pale yellow oil (61% yield): ^{1}H NMR (400 MHz, $CDCl_3$) δ 7.30-7.24 (m, 2H), 7.14-7.06 (m, 3H), 5.36 (s, 1H), 3.79 (s, 3H); ^{13}C NMR (100 MHz, $CDCl_3$) δ 168.8, 135.7, 129.3, 128.8, 128.6, 128.4, 127.2, 53.4, 46.5.

Methyl 2-bromo-2-(4-fluorophenyl)acetate (2l)[7]

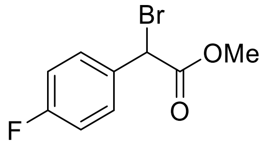

Following the general procedure for the C(sp^3)–H Bromination; purified by column chromatography on silica gel using (20:1 hexane/ethyl acetate; colorless oil (57% yield): ^{1}H NMR (400 MHz, $CDCl_3$) δ 7.57-7.52 (m, 2H), 7.08-7.02 (m, 2H), 5.34 (s, 1H), 3.79 (s, 3H); ^{13}C NMR (100 MHz, $CDCl_3$) δ 168.7, 163.0 (d, J = 248 Hz), 131.6 (d, J = 3.8 Hz), 130.6 (d, J = 8.6 Hz), 115.8 (d, J = 21.0 Hz), 53.4, 45.3; ^{19}F NMR (376 MHz, $CDCl_3$) δ -111.3.

Methyl 2-bromo-2-(4-bromophenyl)acetate (2m)[7]

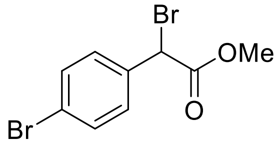

Following the general procedure for the C(sp^3)–H Bromination; purified by column chromatography on silica gel using (20:1 hexane/ethyl acetate; colorless oil (55% yield): ^{1}H NMR (400 MHz, $CDCl_3$) δ 7.50 (d, J = 8.8 Hz, 2H), 7.42 (d, J = 8.8 Hz, 2H), 5.30 (s, 1H), 3.79 (s, 3H); ^{13}C NMR (100 MHz, $CDCl_3$) δ 168.4, 134.7, 132.0, 130.3, 123.6, 53.5, 45.3.

4-Bromo-4-methylpentan-2-yl 4-(trifluoromethyl)benzoate (2n)

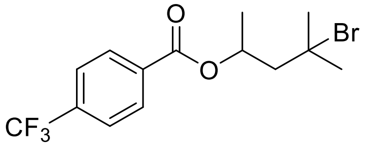

^{1}H NMR (400 MHz, $CDCl_3$) δ 8.15 (d, J = 8.2 Hz, 2H), 7.71 (d, J = 8.2 Hz, 2H), 5.56-5.49 (m, 1H), 2.39 (dd, J = 15.6, 8.7 Hz, 1H), 2.22 (dd, J = 15.6, 2.3 Hz, 1H), 1.83 (s, 3H), 1.77 (s, 3H), 1.41 (d, J = 6.4 Hz, 3H); ^{13}C NMR (100 MHz, $CDCl_3$) δ 164.6, 134.4 (q, *J* = 32.6 Hz), 133.6, 129.9, 125.4 (q, *J* = 2.9 Hz), 123.6 (q, *J* = 271 Hz), 70.8, 64.3, 52.6, 35.4, 33.8, 21.6; ^{19}F NMR (376 MHz, $CDCl_3$) δ -63.0; HRMS (ESI^+) *m*/*z*: [M^+] Calcd. For $C_{14}H_{17}BrF_3O_2$: 353.0364; Found 353.0366.

3-Bromo-3-methylbutyl 2-(4-fluorophenyl)acetate (2o)

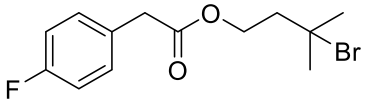

Following the general procedure for the C(sp^3)–H Bromination; purified by column chromatography on silica gel (hexane/ethyl acetate = 10:1); colorless oil (30% yield): ^{1}H NMR (400 MHz, $CDCl_3$) δ 7.23 (dd, *J* = 8.2, 5.5 Hz, 2H), 7.01 (dd, *J* = 8.5, 8.2 Hz, 2H), 4.34 (t, *J* = 6.9 Hz, 2H), 3.59 (s, 2H), 2.13 (t, *J* = 6.9 Hz, 2H), 1.75 (s, 6H); ^{13}C NMR (100 MHz, $CDCl_3$) δ 171.3, 162.0 (d, *J* = 244 Hz), 130.8 (d, *J* = 7.6 Hz), 129.5 (d, *J* = 3.9 Hz), 115.4 (d, *J* = 22.0 Hz), 64.1, 63.1, 45.1, 40.5, 34.6; ^{19}F NMR (376 MHz, $CDCl_3$) δ -115.5; HRMS (ESI^+) *m*/*z*: [M^+] Calcd. for $C_{13}H_{17}BrFO_2$: 303.0396; Found 303.0395.

3-4. Conversion of introduced bromine atom

3-Fluoro-3-methylbutyl 4-(trifluoromethyl)benzoate (3)[8]

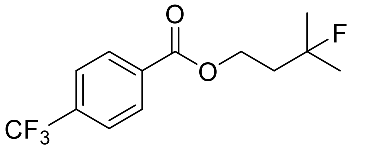

3-Bromo-3-methylbutyl 4-(trifluoromethyl)benzoate (**2a**, 86.0 mg, 0.250 mmol) and Selectfluor (213 mg, 0.600 mmol) were added in a seal tube under argon atmosphere. Freshly distilled dry MeCN (2 mL) was then added. The reaction mixture was stirred at 25 °C for 12 h. Water (5 mL)

was added, and the reaction mixture was extracted with CH_2Cl_2 (3 × 10 mL). The organic phases were combined and dried over anhydrous Na_2SO_4. After the removal of solvent under reduced pressure, the crude product was purified by column chromatography on silica gel (hexane/ethyl acetate = 20:1) as the eluent to give **3** as a colorless oil. Yield: 61.1 mg (86%). ^{1}H NMR (400 MHz, $CDCl_3$) δ 8.14 (d, *J* = 8.2 Hz, 2H), 7.71 (d, *J* = 8.2 Hz, 2H), 4.52 (t, *J* = 6.6 Hz, 2H), 2.13 (dt, *J* = 13.3, 6.6 Hz, 2H), 1.45 (d, *J* = 21.5 Hz, 6H); ^{13}C NMR (100 MHz, $CDCl_3$) δ 165.4, 134.5 (q, *J* = 32.6 Hz), 133.5, 130.1 (q, *J* = 10.6 Hz), 125.5 (q, *J* = 3.8 Hz), 123.6 (q, *J* = 271 Hz), 94.2 (d, *J* = 166 Hz), 61.4 (d, *J* = 5.7 Hz), 39.8 (d, *J* = 32.6 Hz), 27.1 (d, *J* = 24.9 Hz); ^{19}F NMR (376 MHz, $CDCl_3$) δ -63.0 (3F), -138.6 (1F).

3,3-Dimethylhex-5-en-1-yl 4-(trifluoromethyl)benzoate (4)[9]

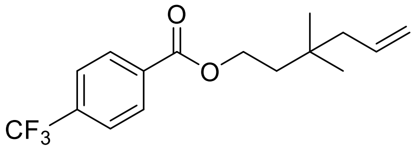

3-Bromo-3-methylbutyl 4-(trifluoromethyl)benzoate (**2a**, 69.1 mg, 0.200 mmol) and allyltributylstannane (132 mg, 0.400 mmol) were added in a reaction tube under argon atmosphere. Then, dry toluene (0.5 mL) and AIBN (azobisisobutyronitrile, 5.0 mg, 15 mmol%) were added and reaction tube was sealed. The mixture was stirred at 80 °C for 8 h. Water (5 mL) was added, and the resulting mixture was extracted with CH_2Cl_2 (3 × 10 mL). The organic phases were combined and dried over anhydrous $MgSO_4$. After the removal of the solvent under reduced pressure, the crude product was purified by column chromatography on silica gel (hexane/ethyl acetate/ (40:1) as the eluent to give **4** as a colorless oil. Yield: 38.3 mg (64%). ^{1}H NMR (400 MHz, $CDCl_3$) δ 8.14 (d, *J* = 8.2 Hz, 2H), 7.70 (d, *J* = 8.2 Hz, 2H), 5.90-5.79 (m, 1H), 5.09-5.02 (m, 2H), 4.42 (t, *J* = 7.4 Hz, 2H), 2.04 (d, *J* = 7.3 Hz, 2H), 1.72 (t, *J* = 7.4 Hz, 2H), 0.98 (s, 6H); ^{13}C NMR (100 MHz, $CDCl_3$) δ 165.4, 134.9, 134.3 (q, *J* = 32.6 Hz), 133.6, 129.9, 125.3 (q, *J* = 3.8 Hz), 123.6 (q, *J* = 272 Hz), 117.5, 62.9, 46.8, 39.5, 32.6, 27.1; ^{19}F NMR (376 MHz, $CDCl_3$) δ -63.0; HRMS (ESI$^+$) *m/z*: [M] Calcd. for $C_{16}H_{20}F_3O_2$, 301.1415; Found 301.1415.

4. References

1. Guo, S.; Zhang, X.; Tang, P. *Angew. Chem. Int. Ed.* **2015**, *54*, 4065-4069.
2. Wang, X.; Wang, S.; Xue, W.; Gong, H. *J. Am. Chem. Soc.* **2015**, *137*, 11562-11565.
3. Inomata, S.; Harada, Y.; Matsuoka, S.; Ishizone, T. *Tetrahedron* **2013**, *69*, 3238-3248.
4. Khusnutdinov, R. I.; Shchadneva, N. A.; Khisamova, L. F. *Russ. J. Org. Chem.* **2015**, *51*, 184-187.
5. Senchyk, G. A., Lysenko, A. B., Boldog, I., Rusanov, E. B., Chernega, A. N., Krautscheid, H., Domasevitch K. V. *Dalton Trans.* **2012**, *41*, 8675-8689.
6. Berndt, J.-P.; Engel, A.; Hrdina, R.; Dehnen, S.; Schreiner, P. R. *Organometallics* **2019**, *38*, 329-335.
7. Wang, H.; Sun, X.; Hu, M.; Zhang, X.; Xie, L.; Gu, S. *Adv. Synth. Catal.* **2020,** *362*, 3347-3351.
8. Chen, H.; Liu, Z.; Lv, Y.; Tan, X.; Shen, H.; Yu, H. Z.; Li, C. *Angew. Chem. Int. Ed.* **2017**, *56*, 15411-15415.
9. Keck, G. E.; Yates, J. B. *J. Am. Chem. Soc.* **1982**, *104*, 5829-5831.

5. ^{1}H and ^{13}C NMR spectra

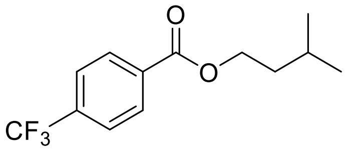

1a

^{1}H NMR

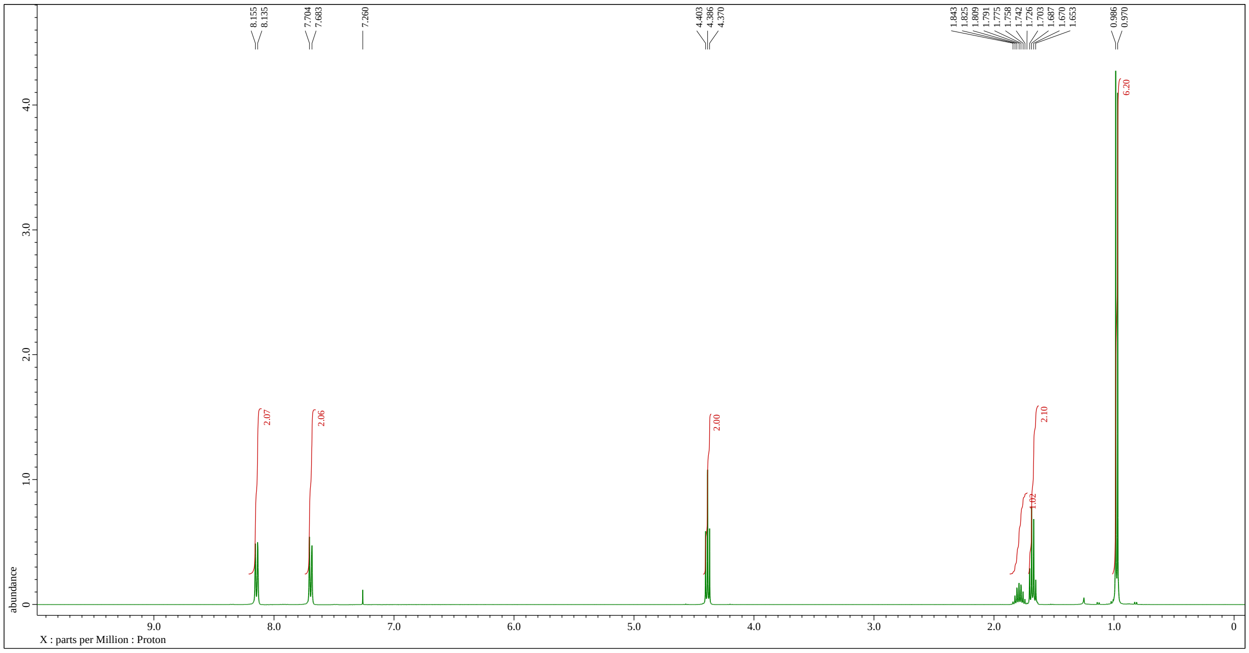

^{13}C NMR

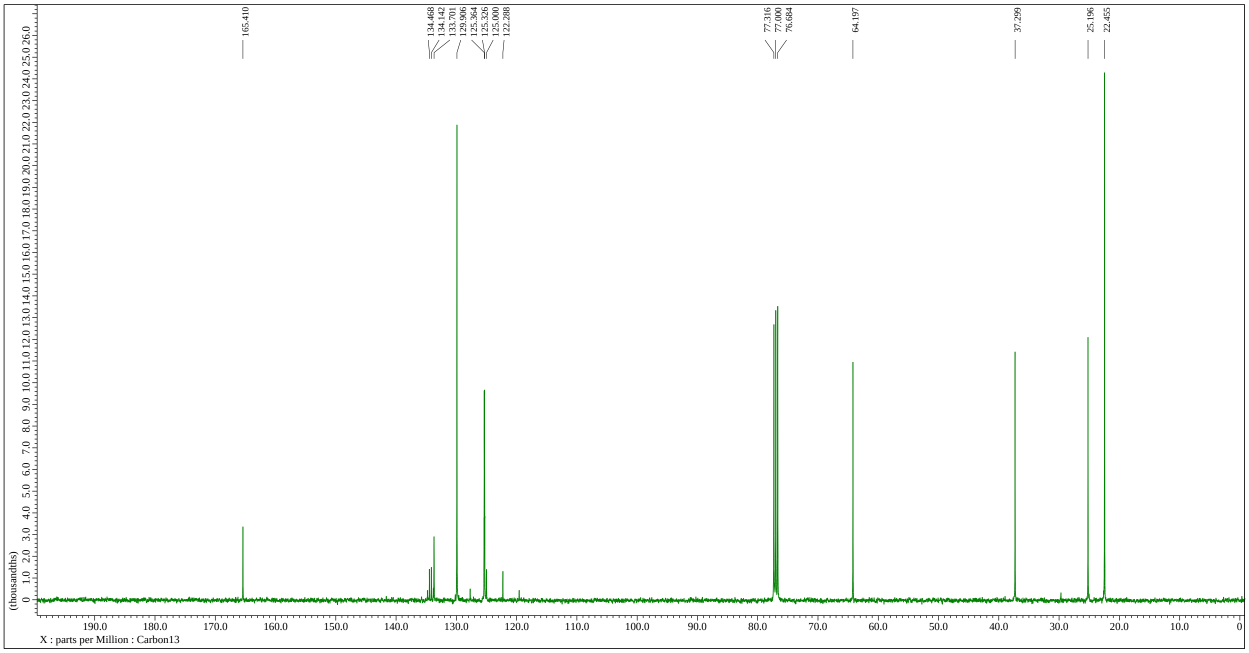

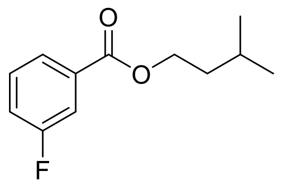

1c

^{1}H NMR

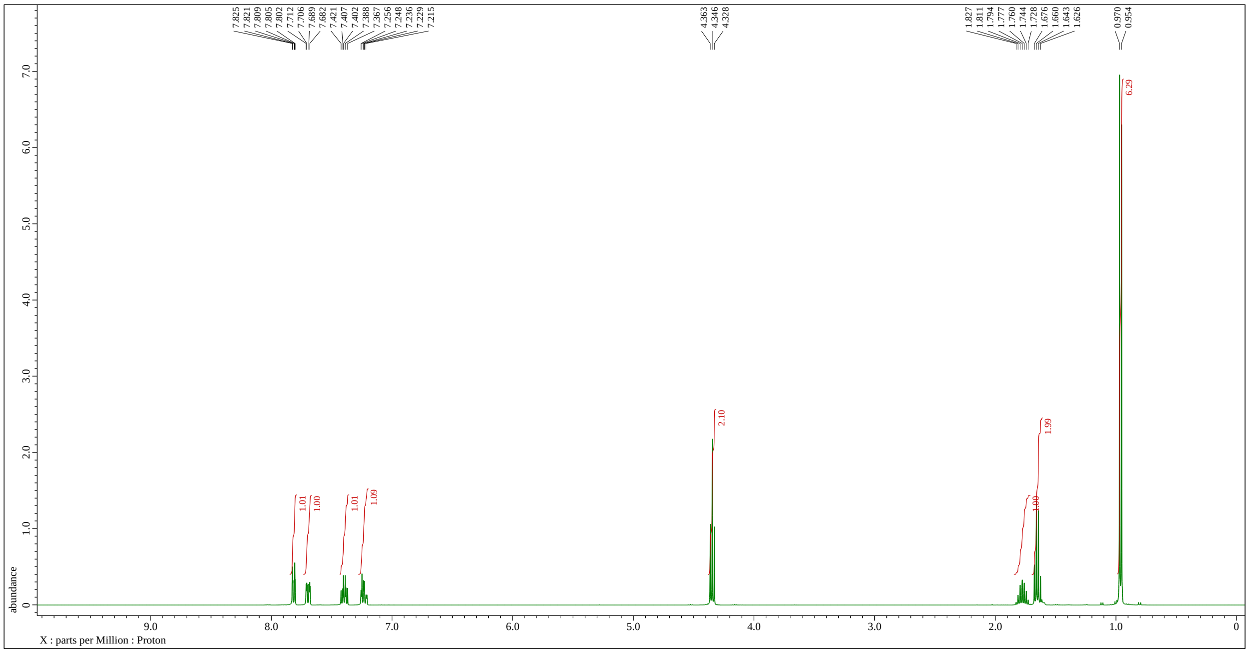

^{13}C NMR

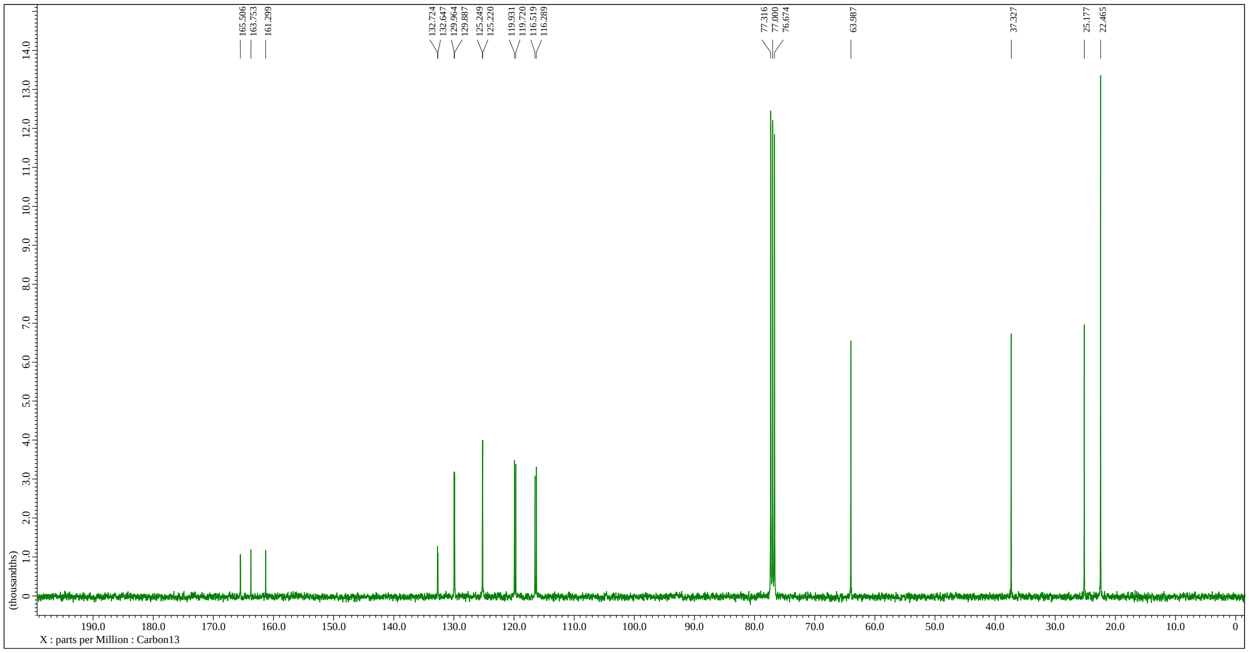

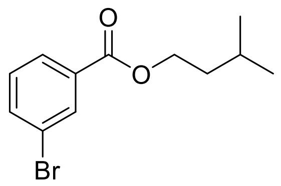

1e

^{1}H NMR

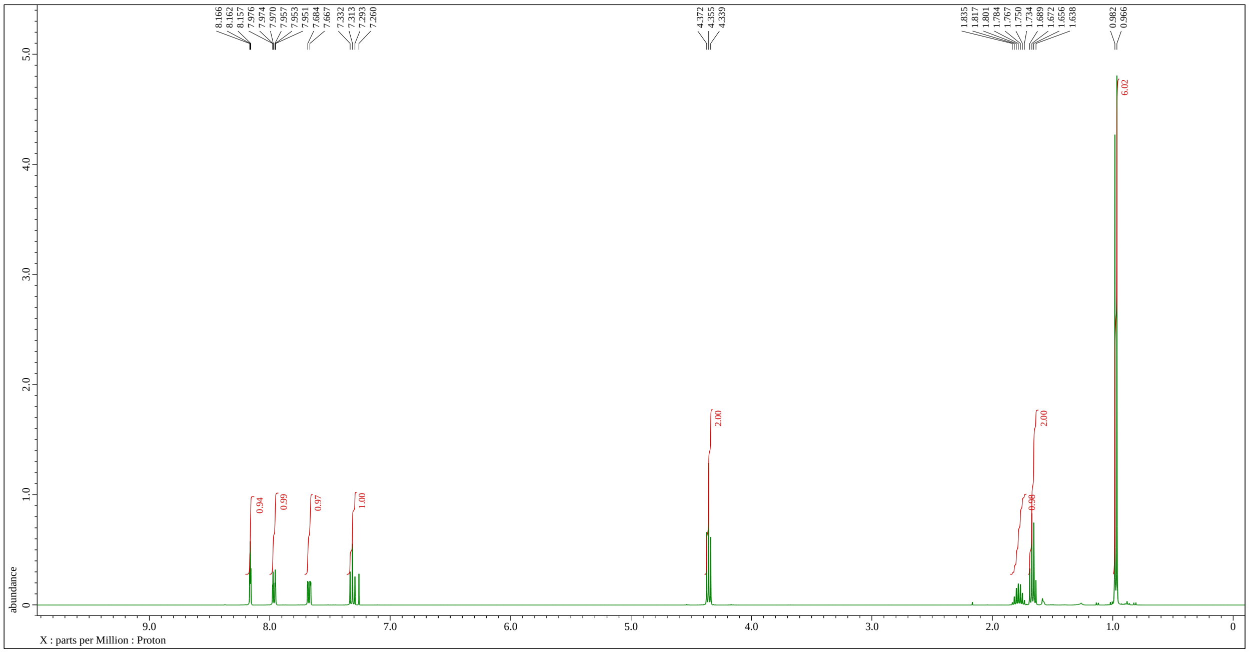

^{13}C NMR

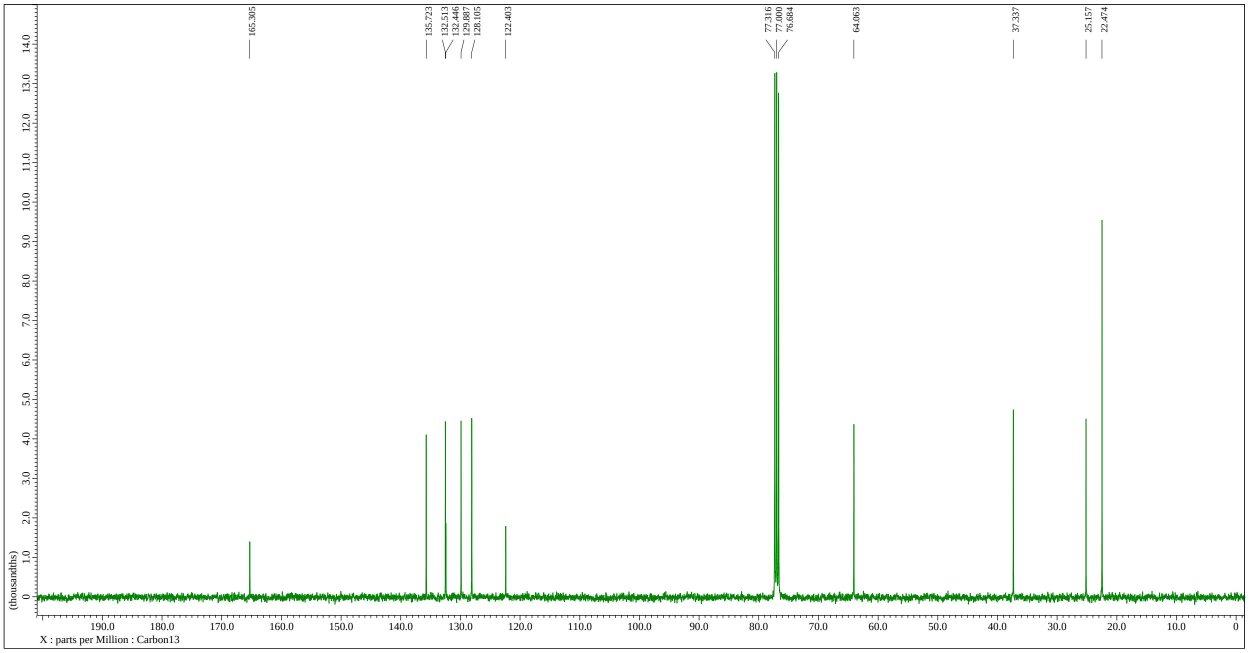

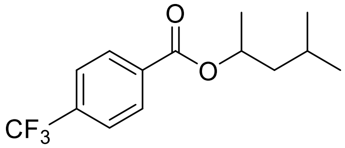

1o

^{1}H NMR

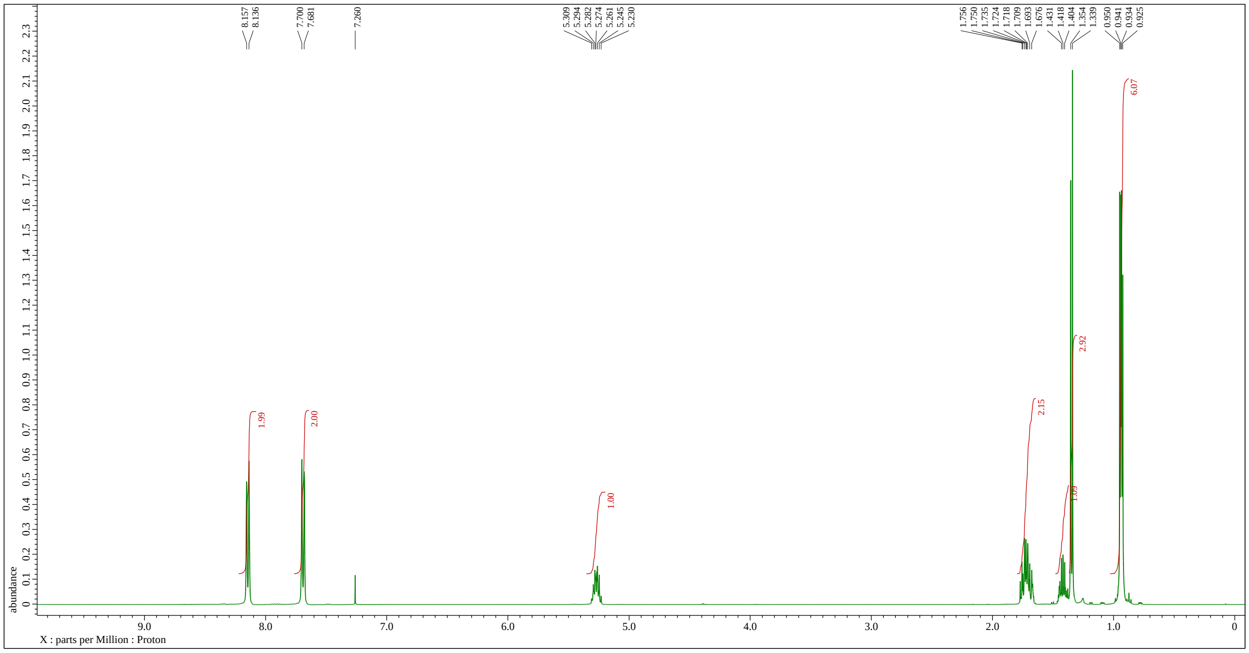

^{13}C NMR

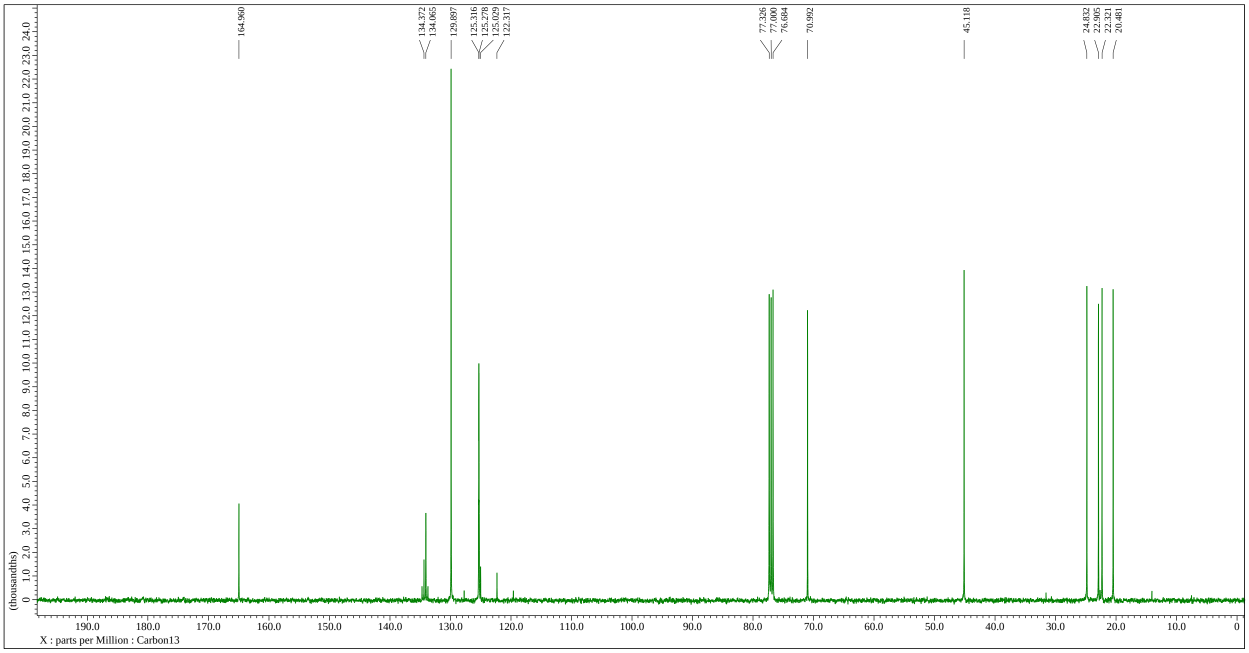

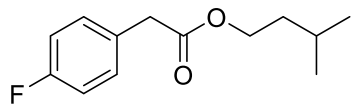

1p

^{1}H NMR

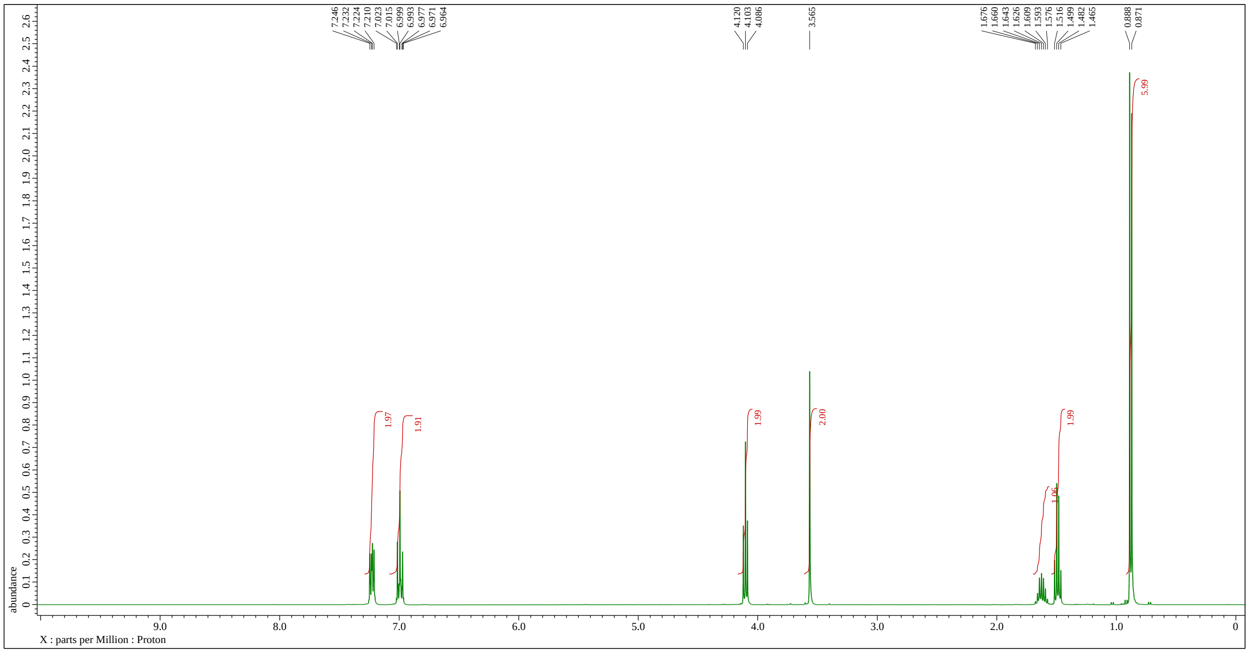

^{13}C NMR

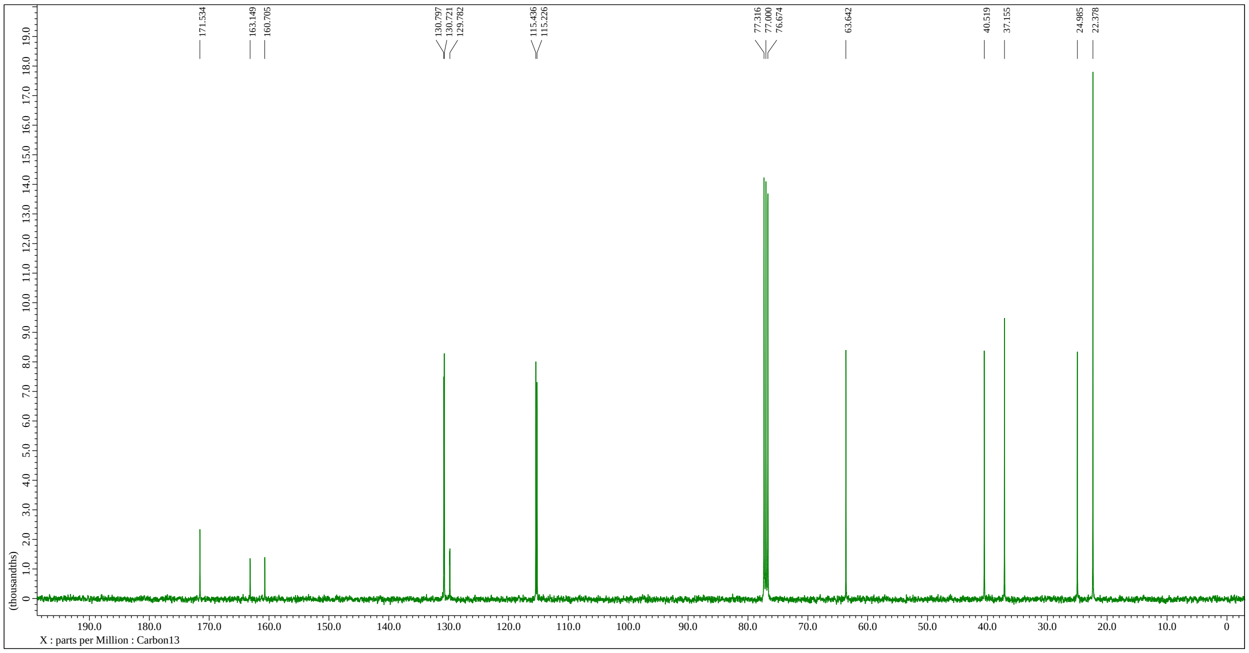

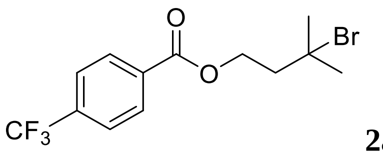

2a

^{1}H NMR

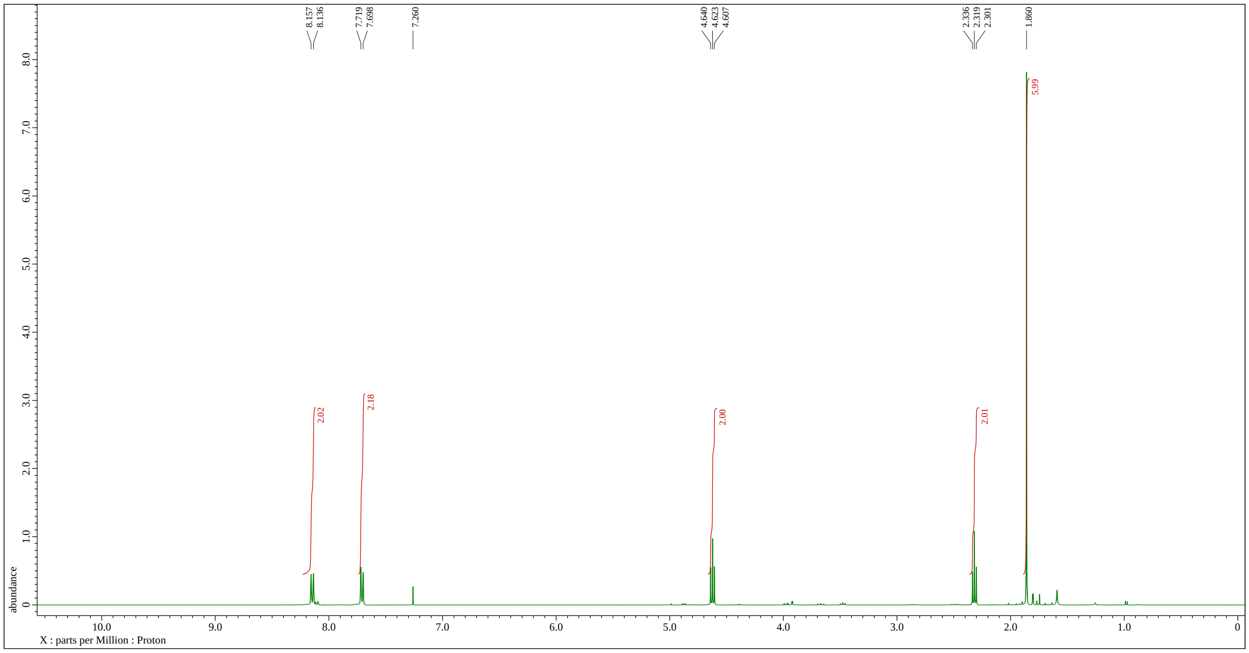

^{13}C NMR

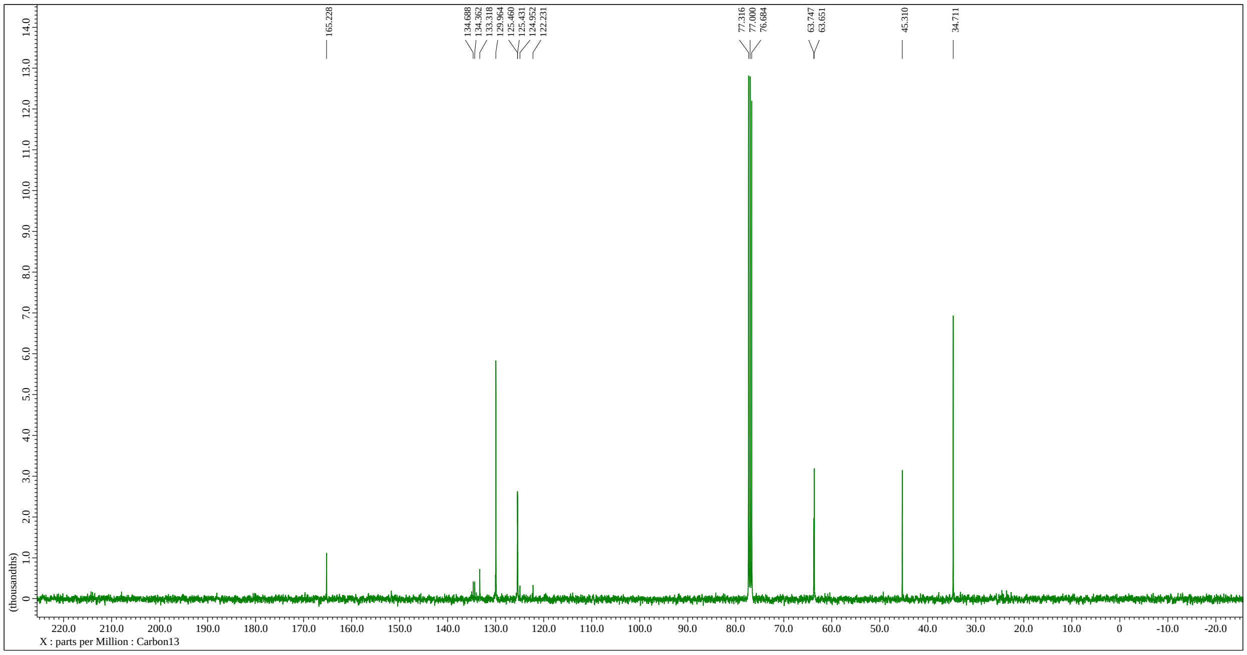

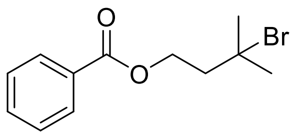

2b

^{1}H NMR

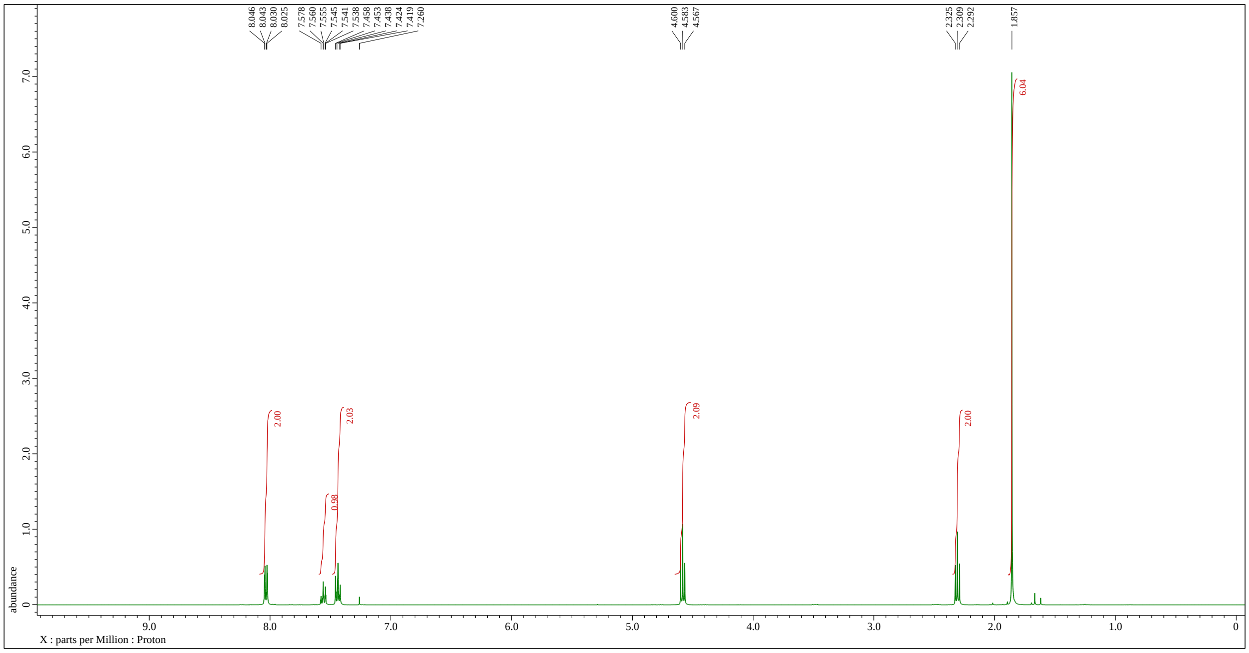

^{13}C NMR

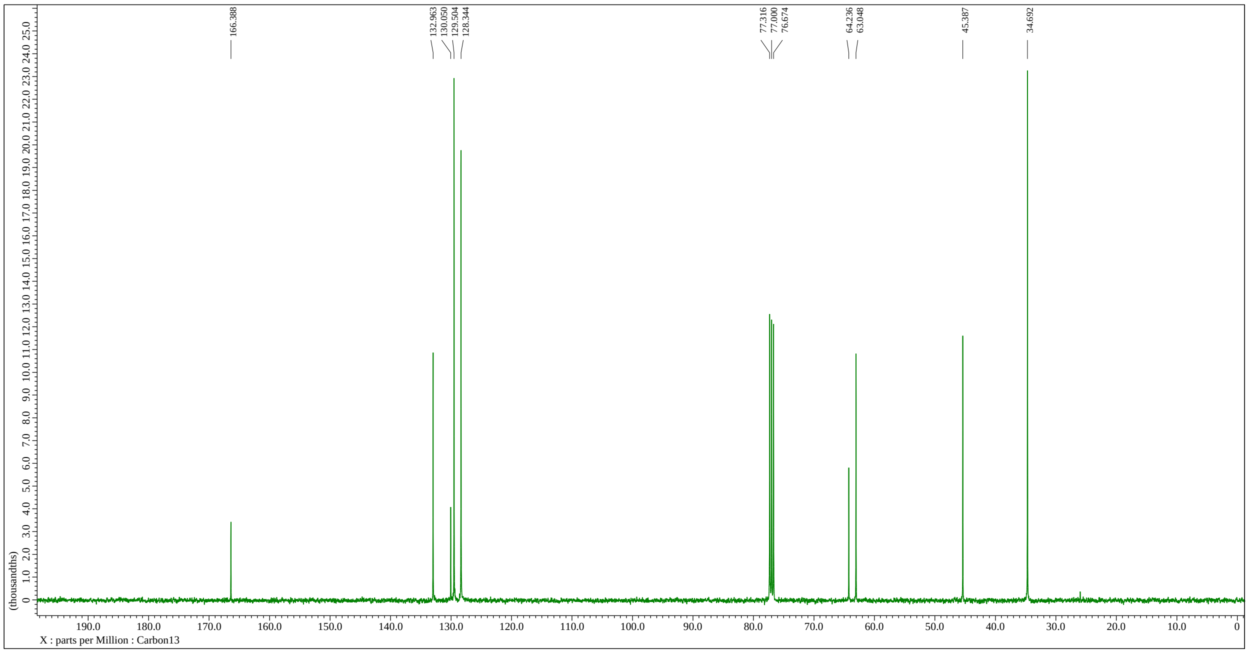

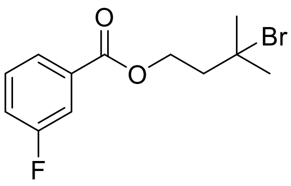

2c

^{1}H NMR

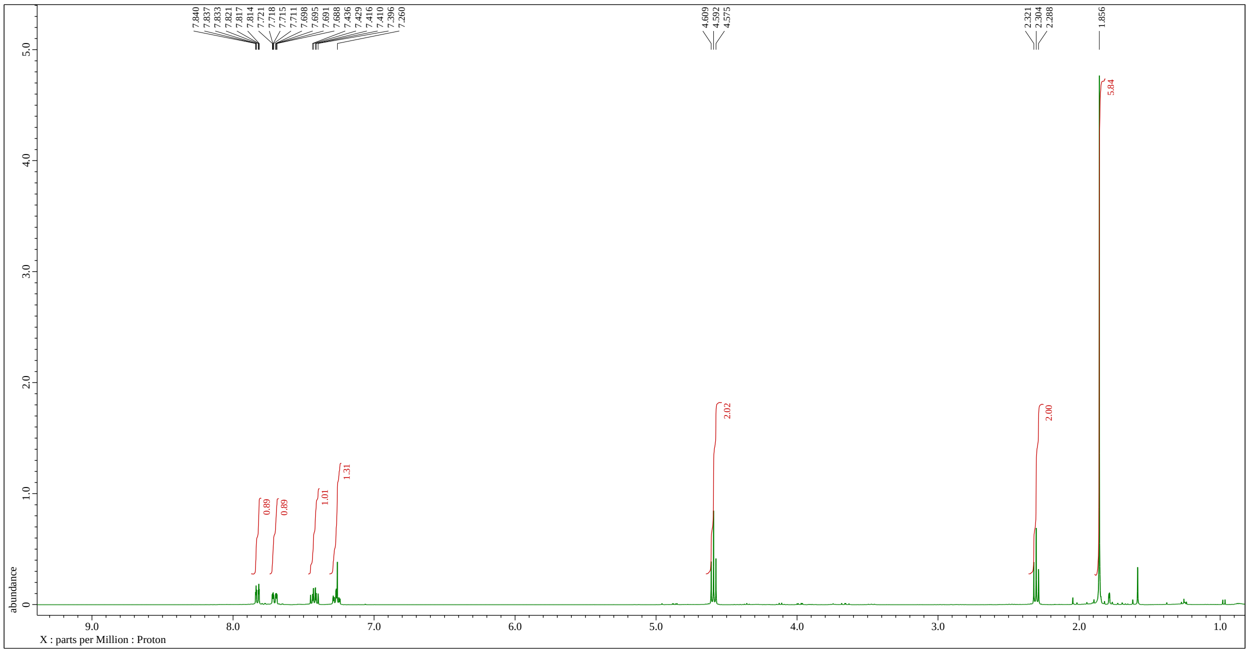

^{13}C NMR

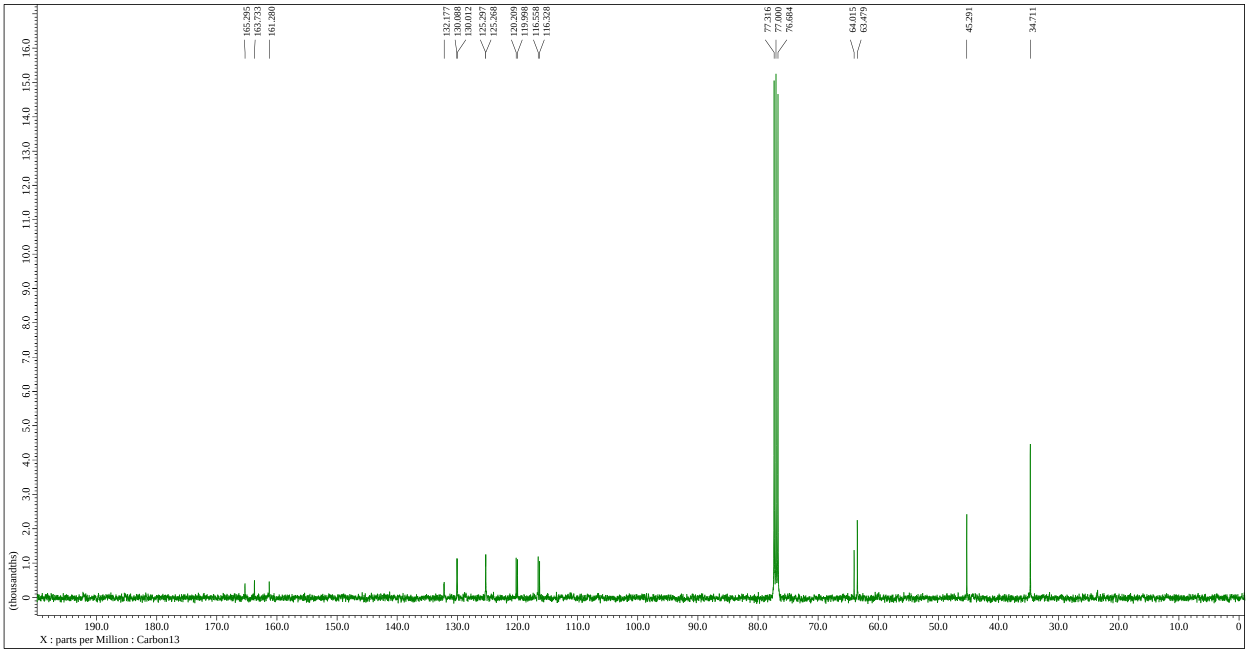

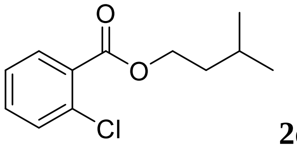

2d

^{1}H NMR

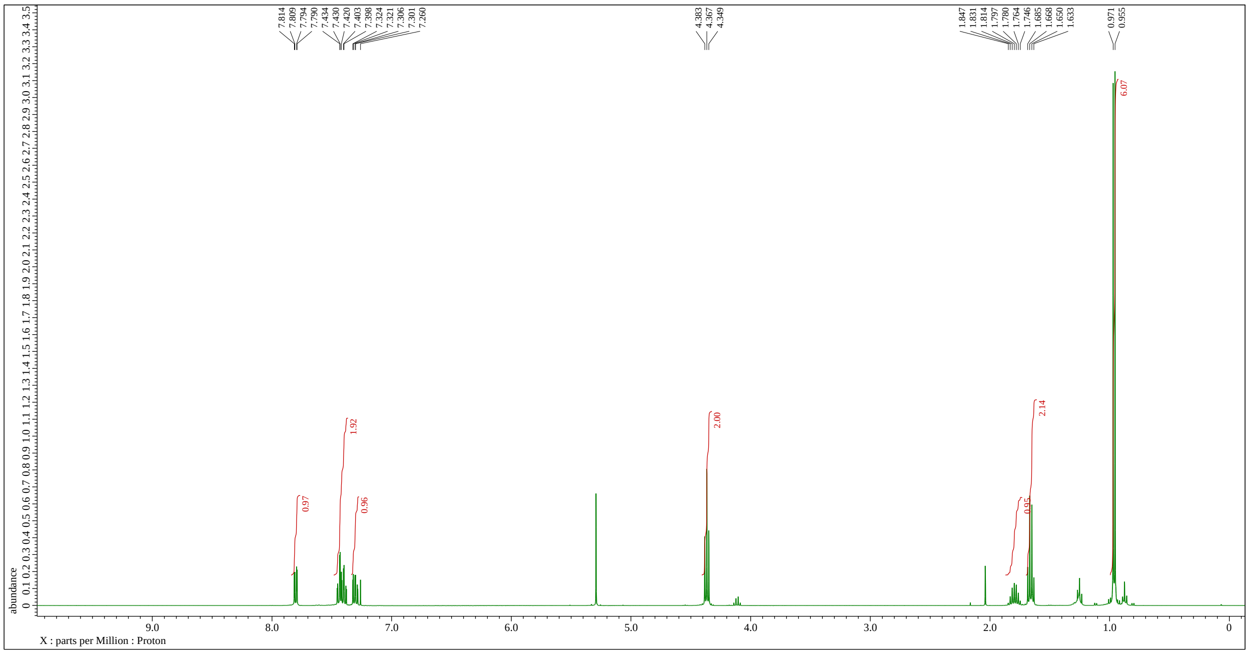

^{13}C NMR

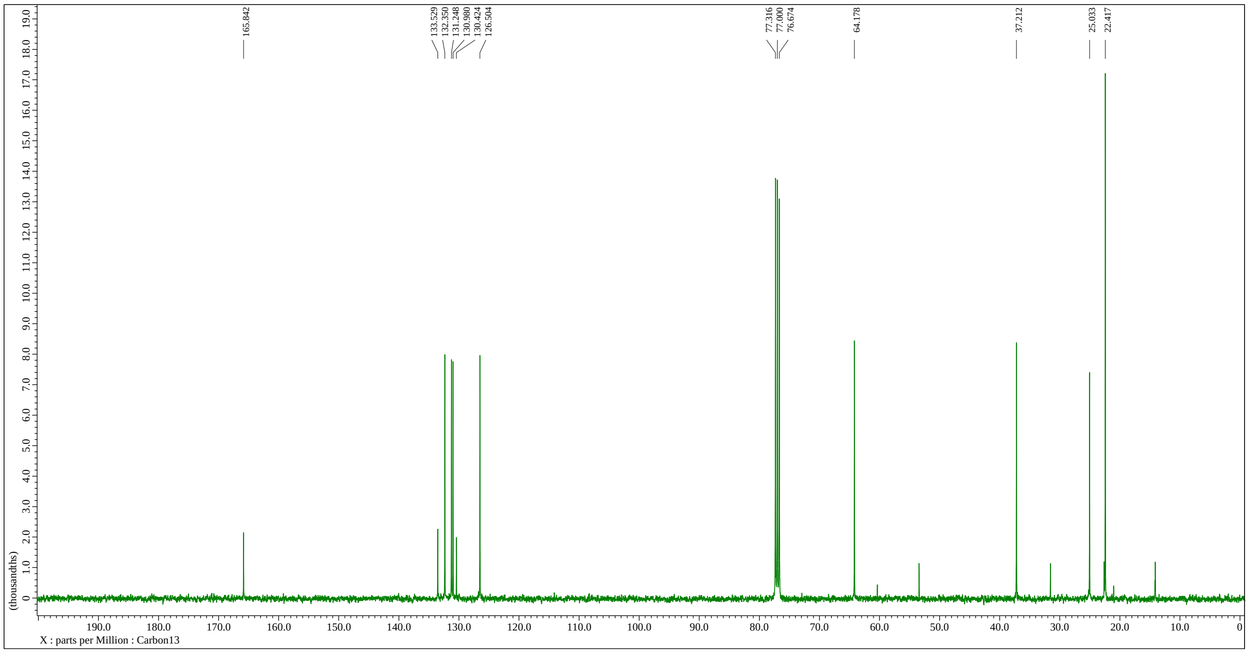

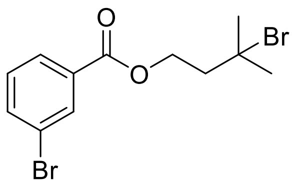

2e

^{1}H NMR

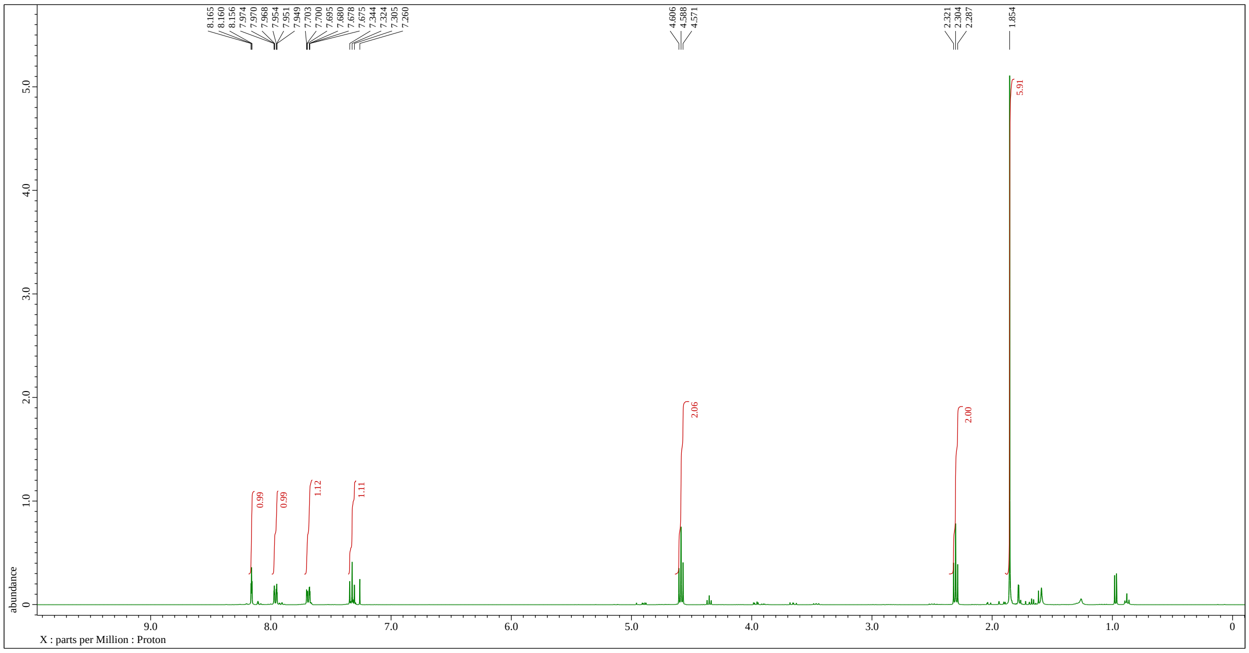

^{13}C NMR

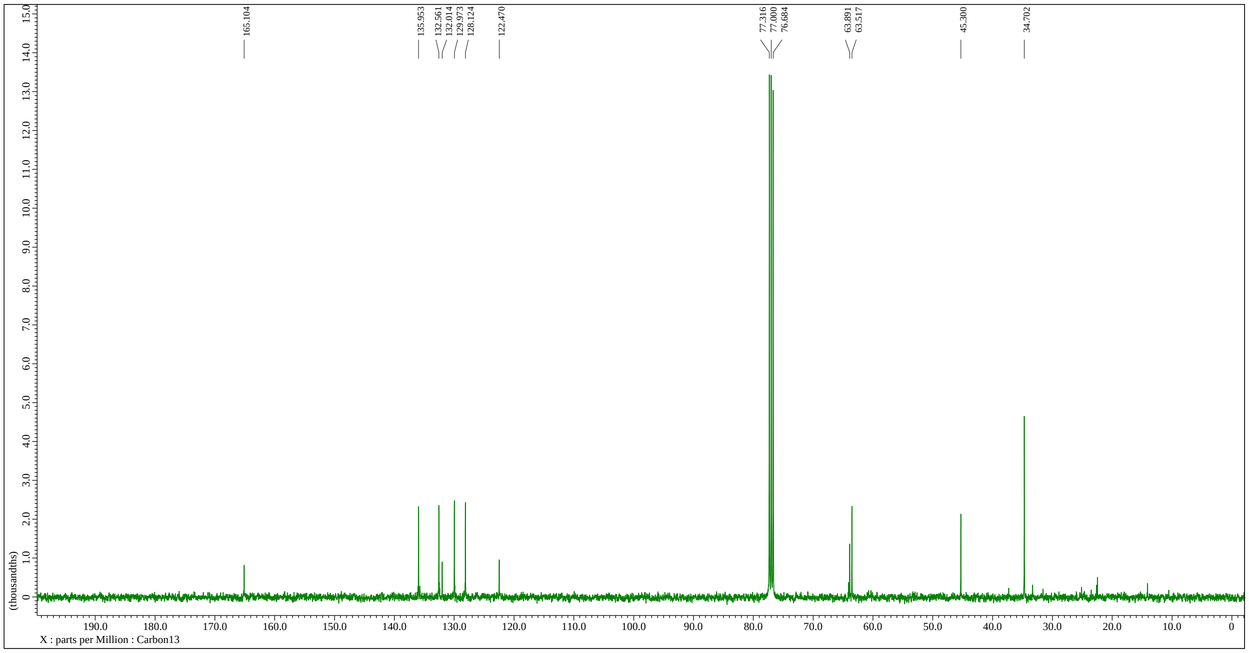

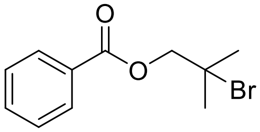

2f

^{1}H NMR

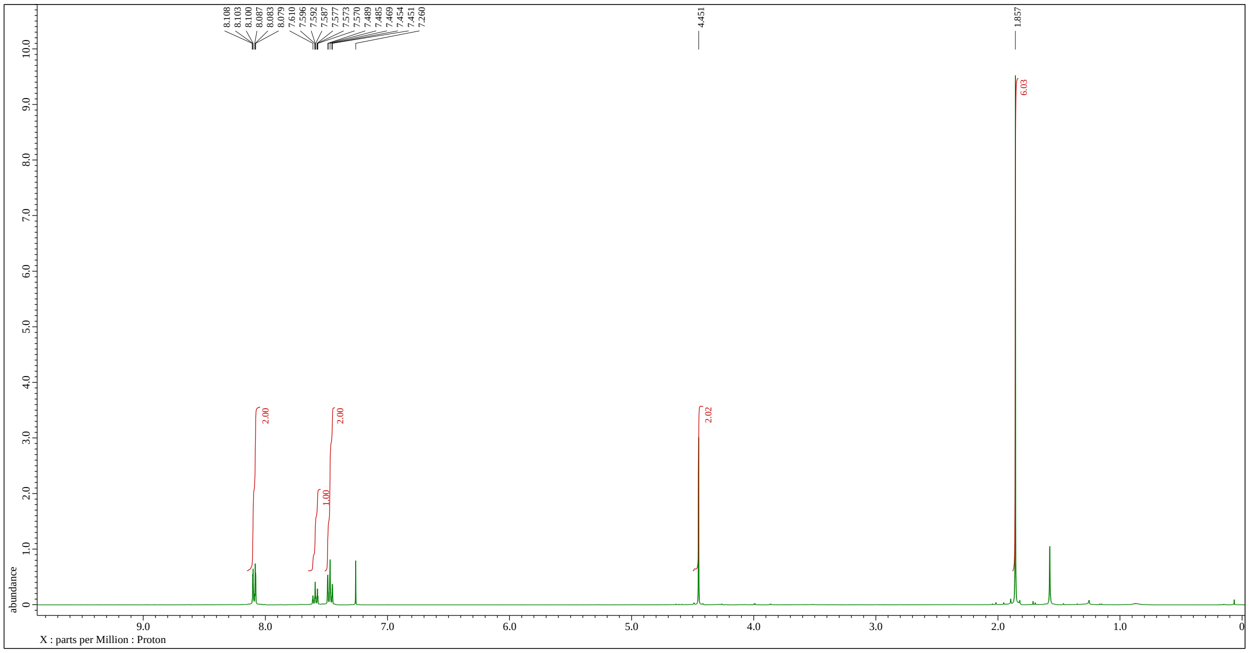

^{13}C NMR

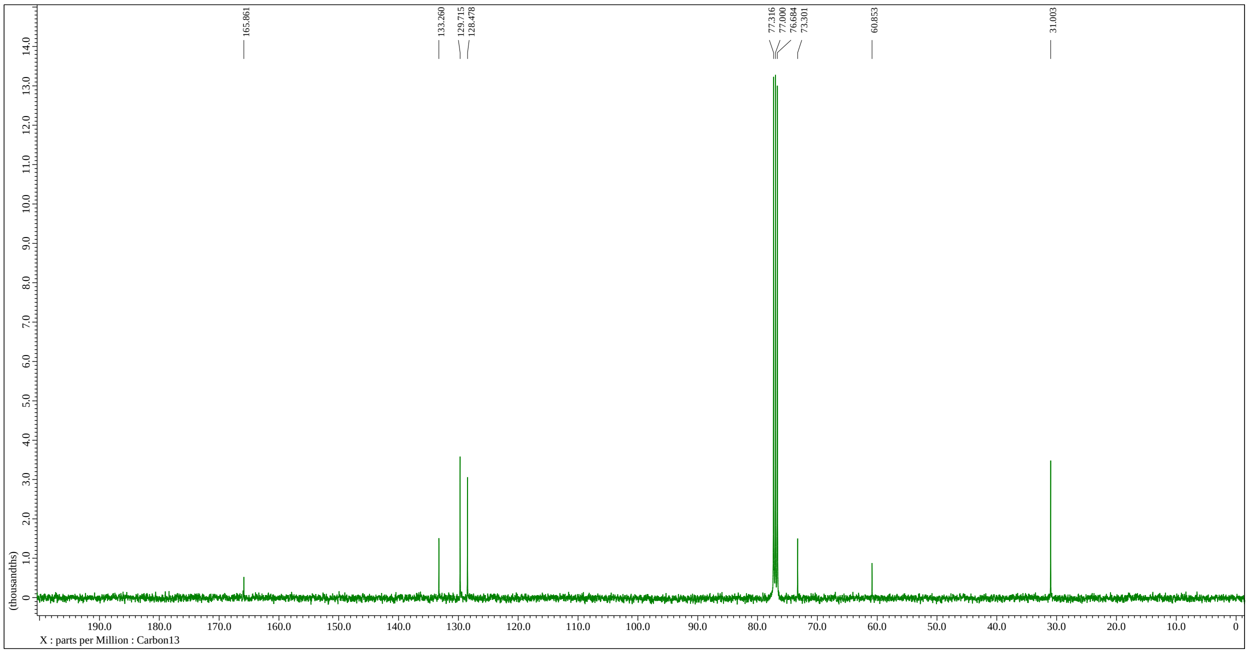

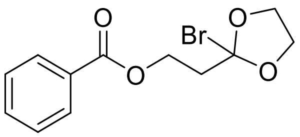

2g

^{1}H NMR

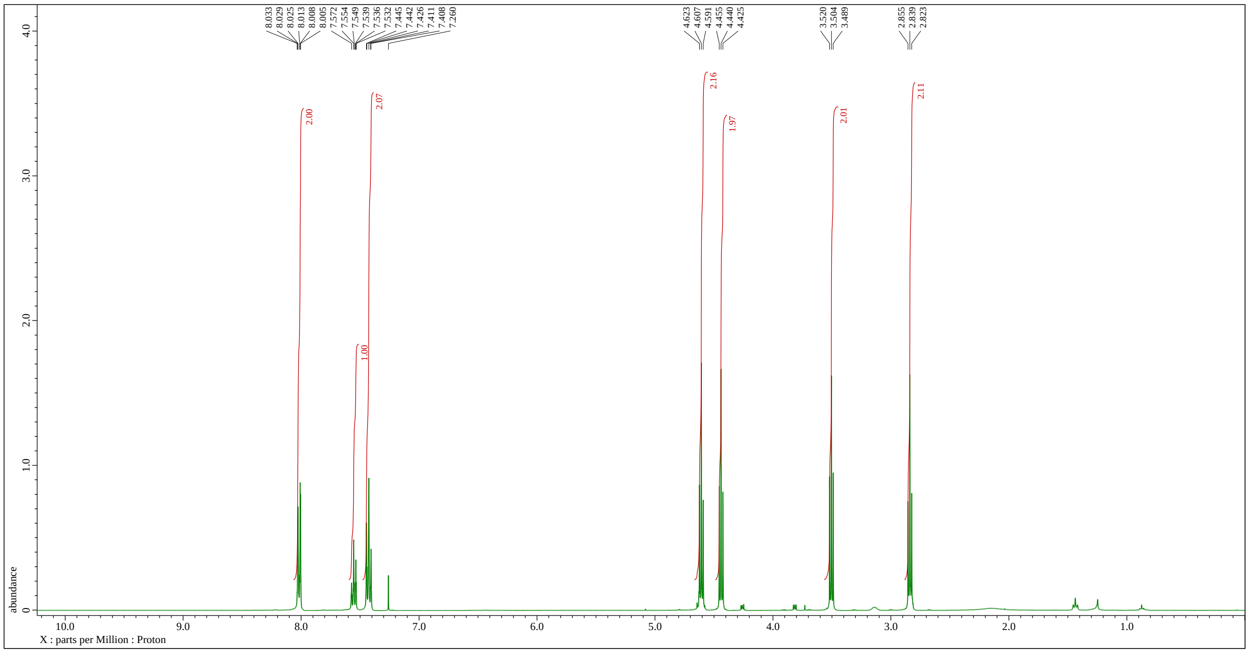

^{13}C NMR

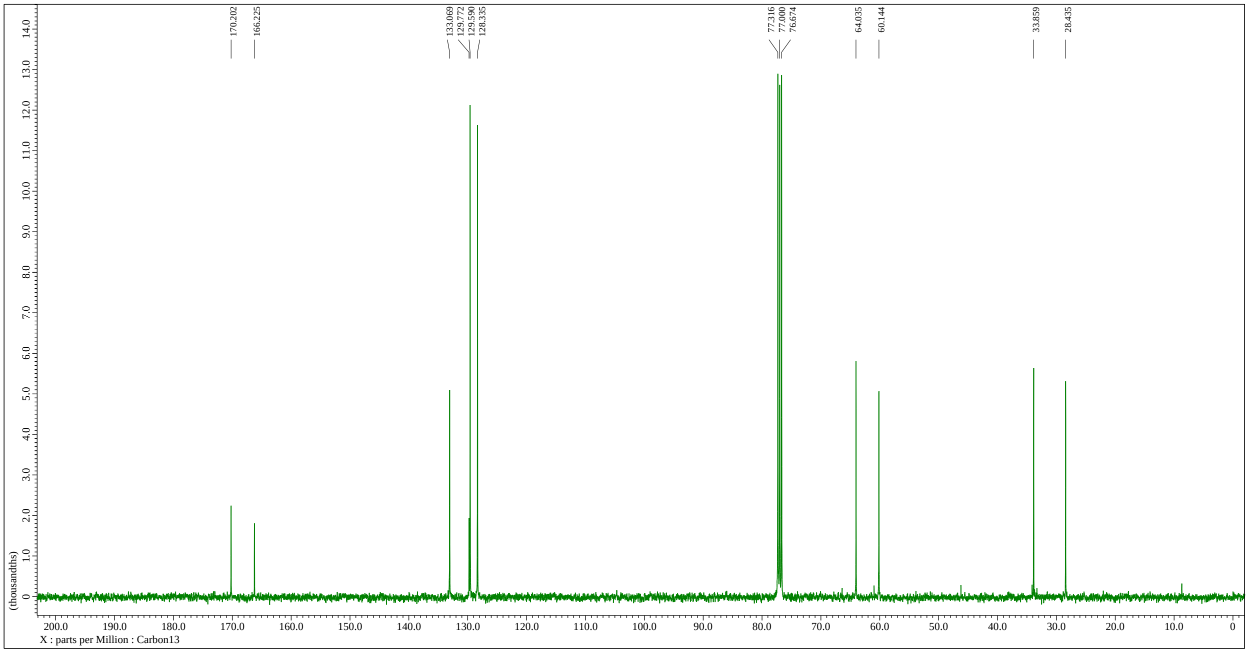

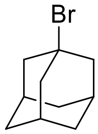

2h

^{1}H NMR

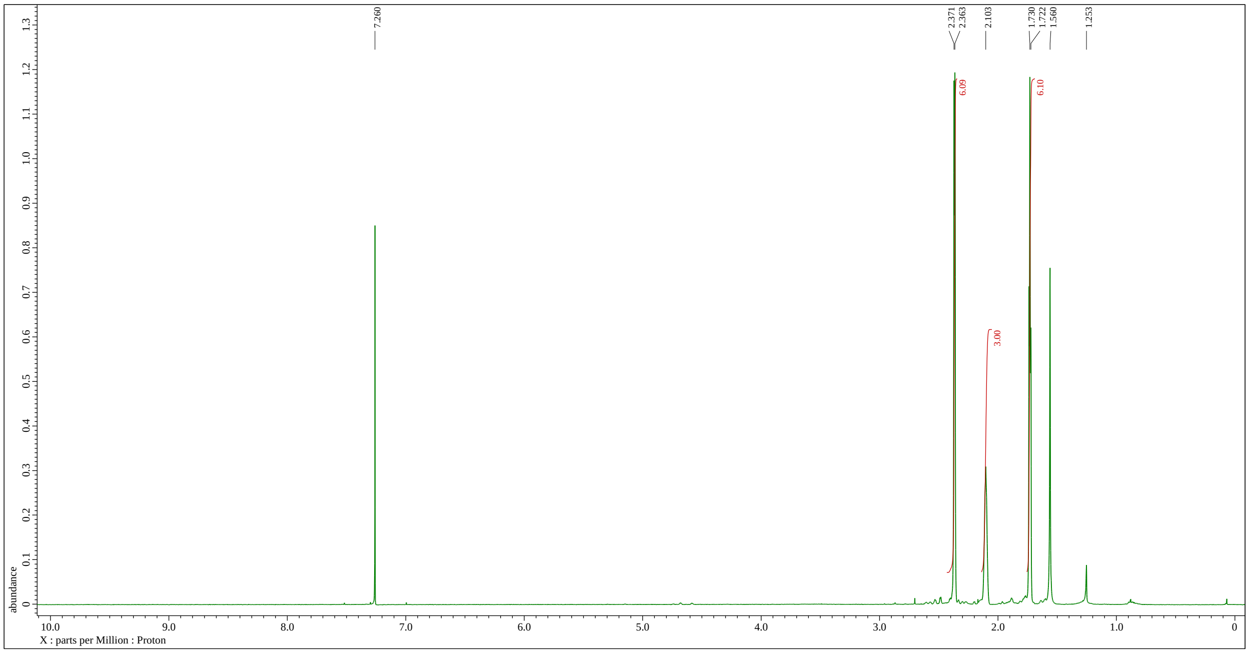

^{13}C NMR

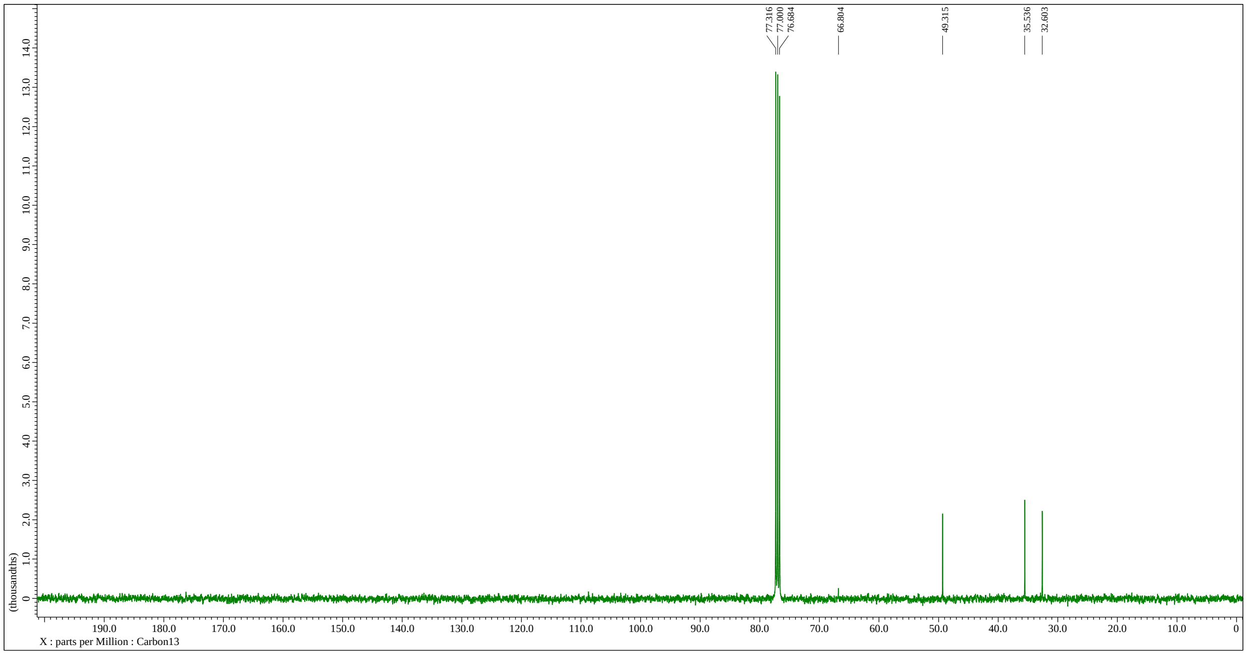

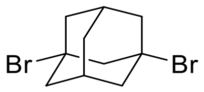

2i'

2h′

^{1}H NMR

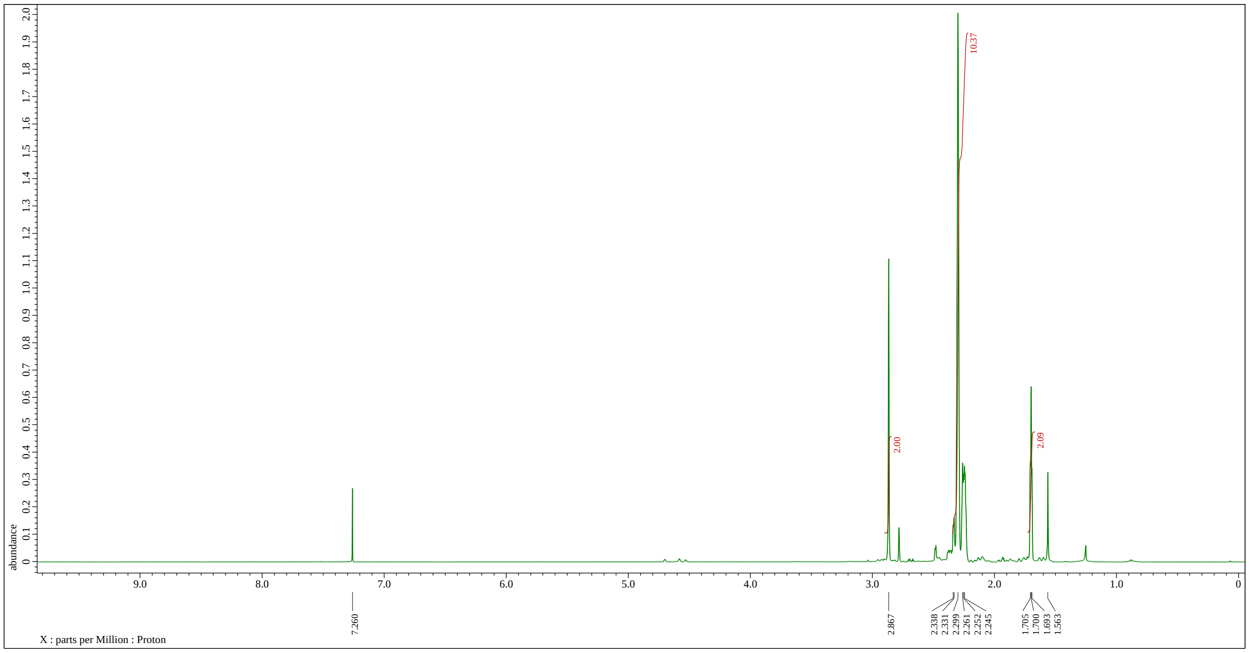

^{13}C NMR

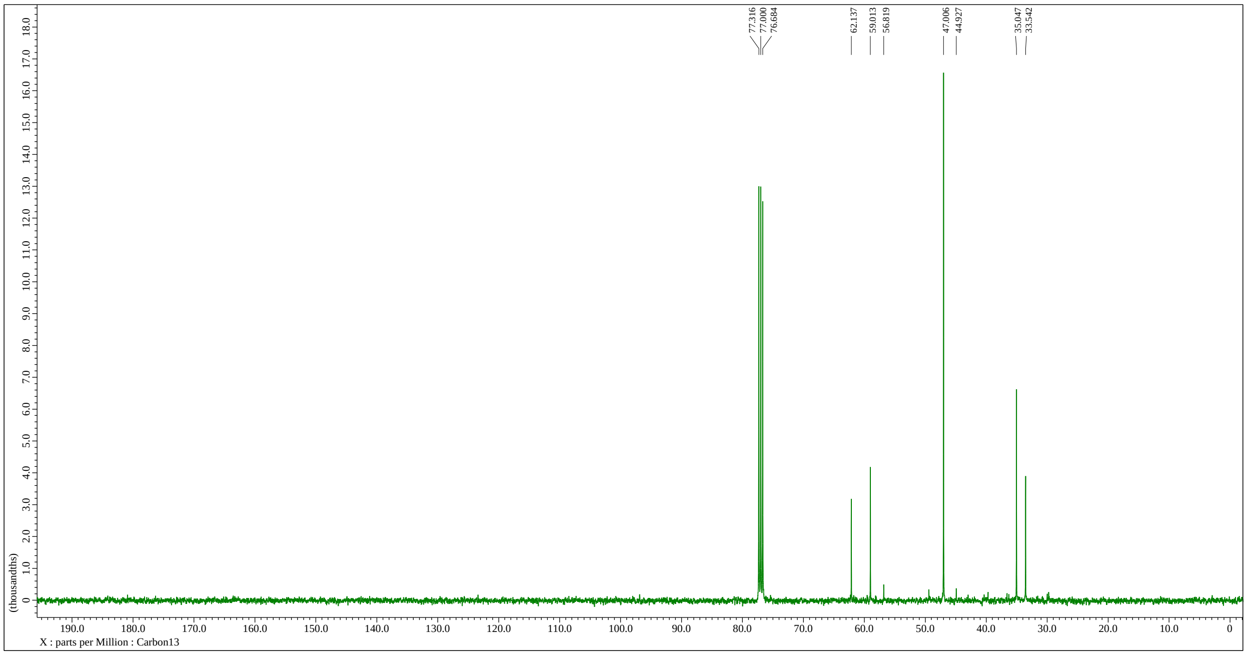

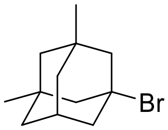

2i

^{1}H NMR

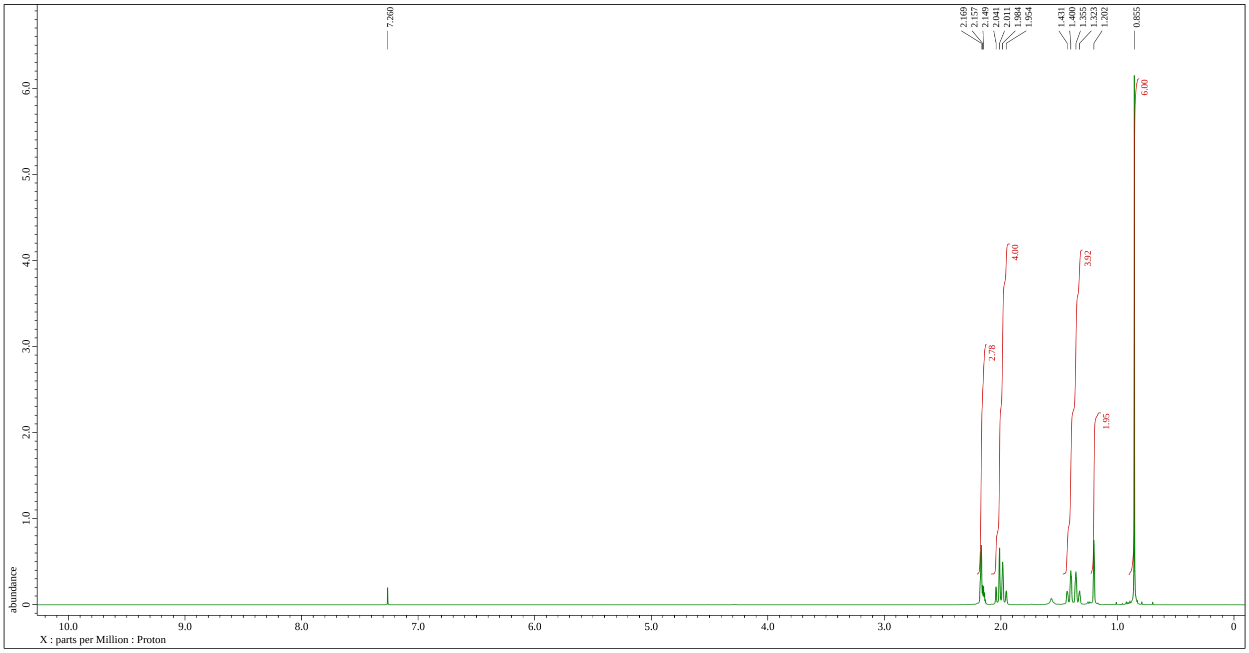

^{13}C NMR

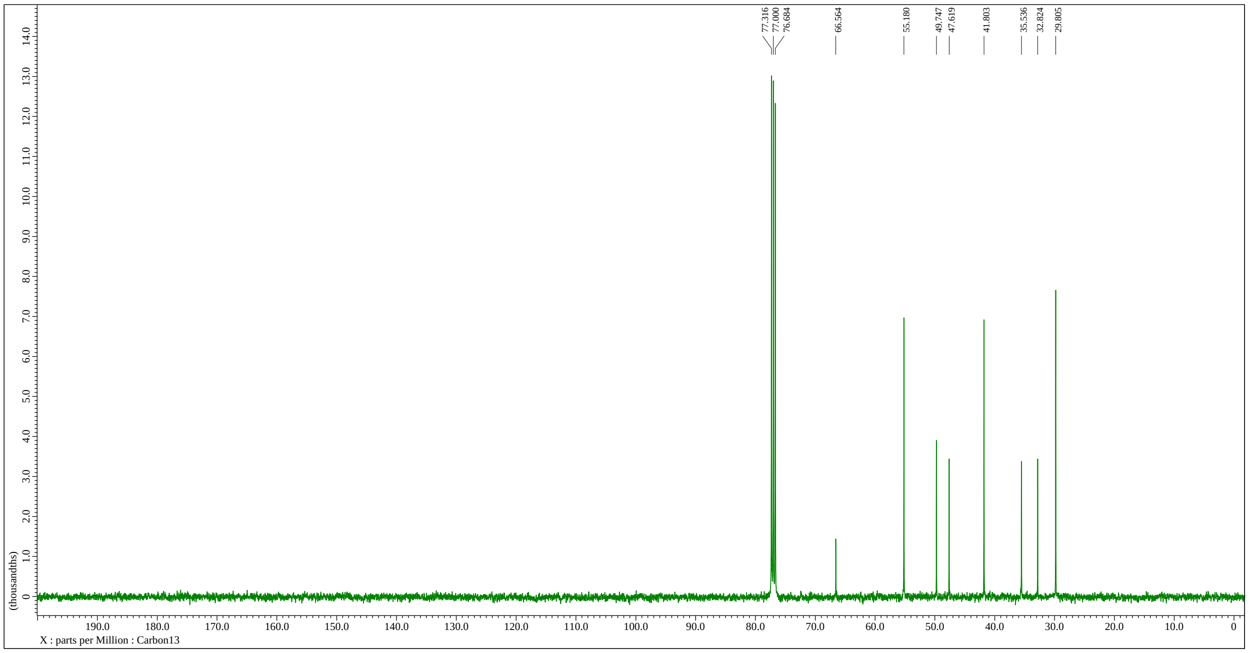

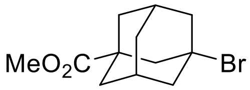

2j

^{1}H NMR

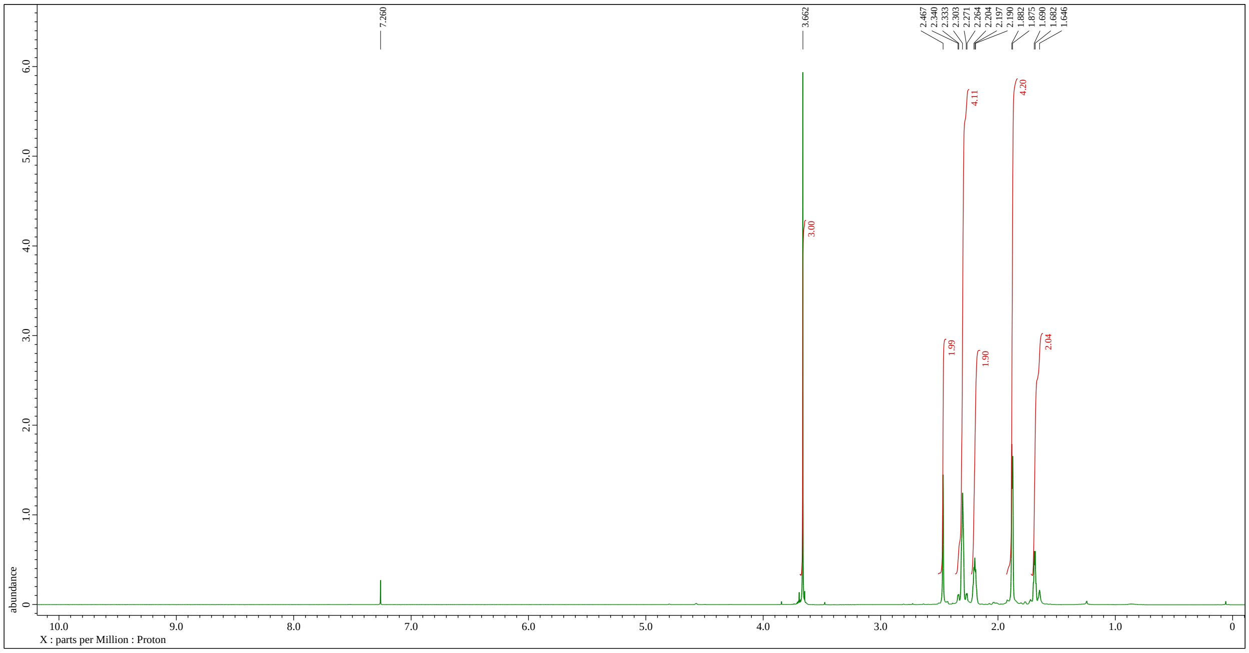

^{13}C NMR

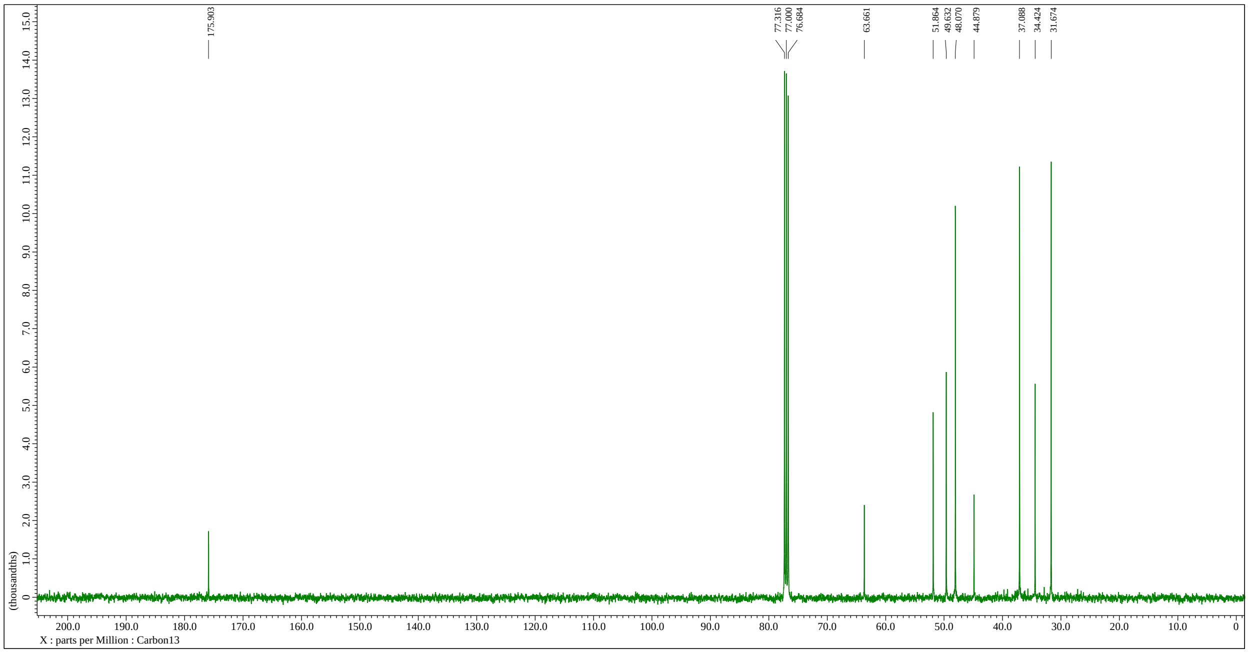

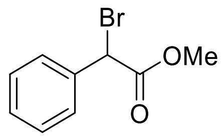

2k

^{1}H NMR

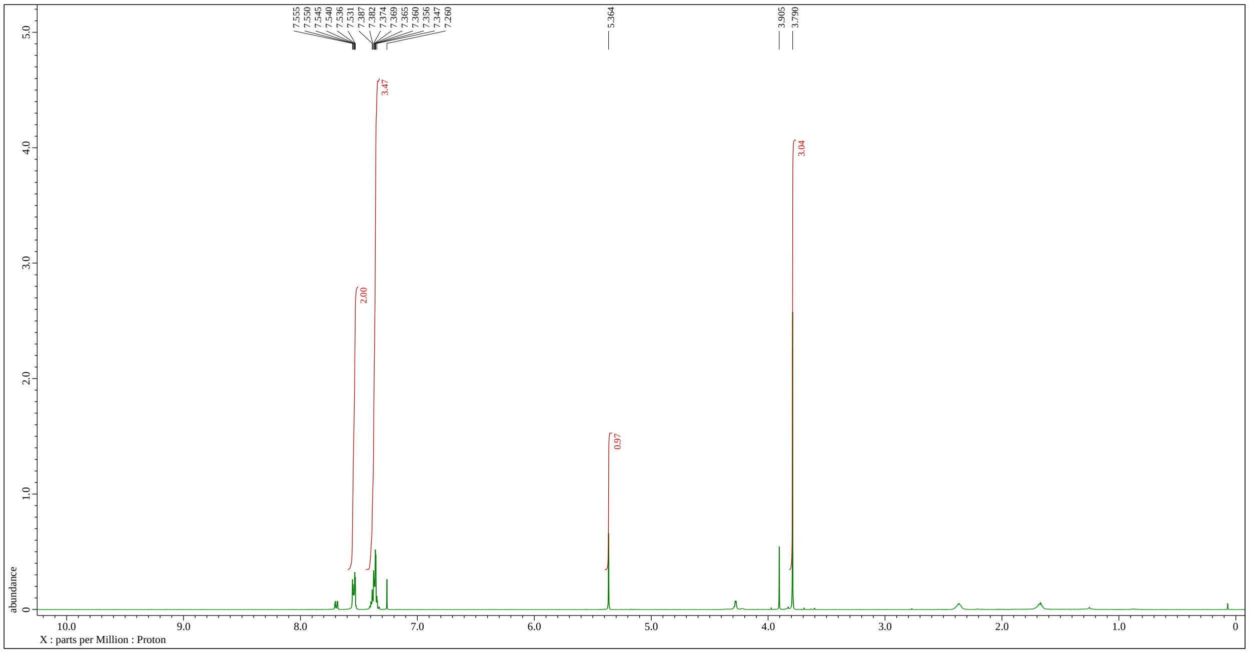

^{13}C NMR

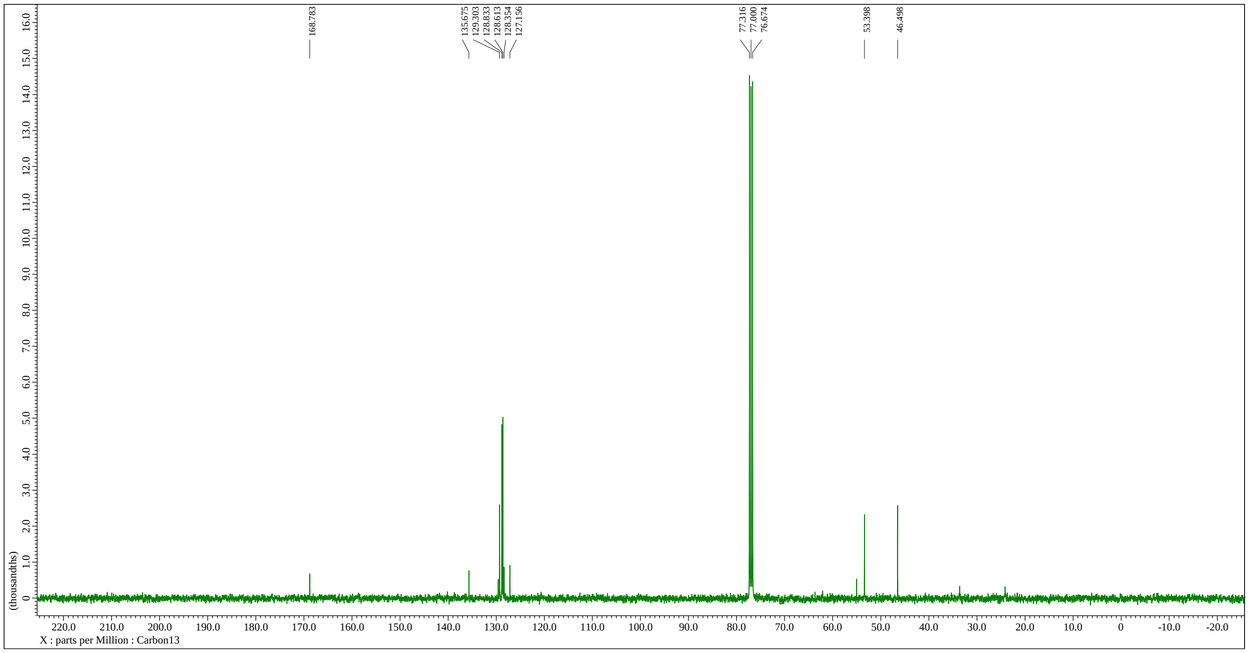

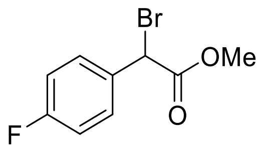

2l

^{1}H NMR

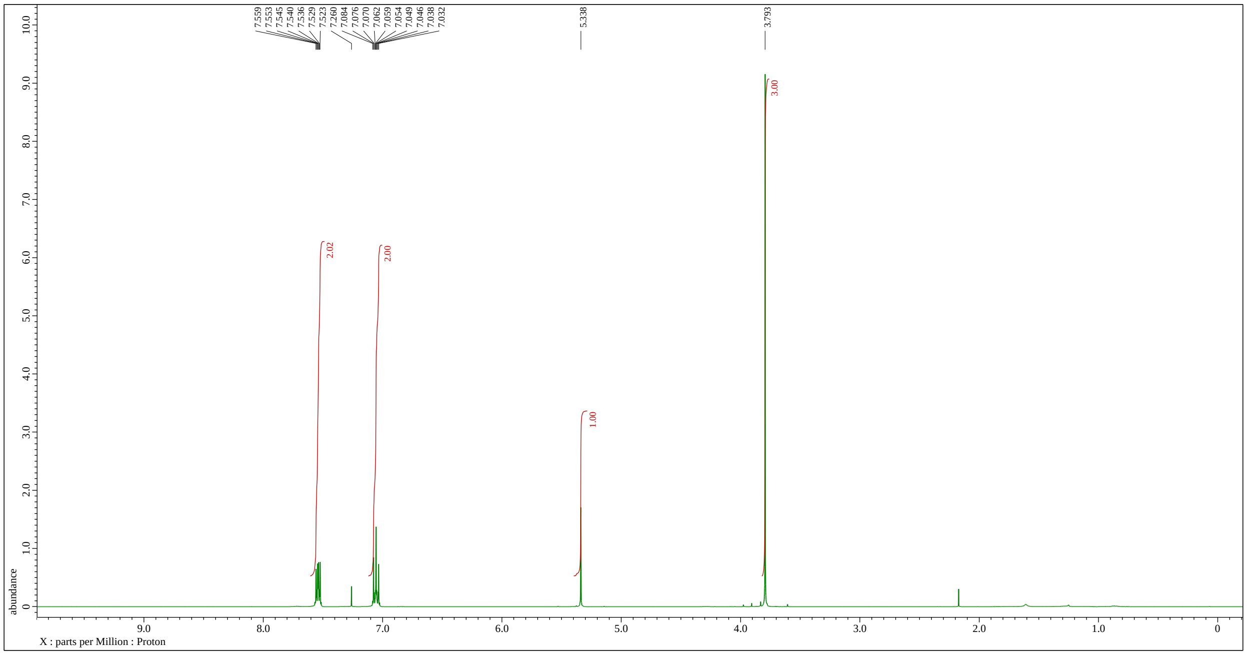

^{13}C NMR

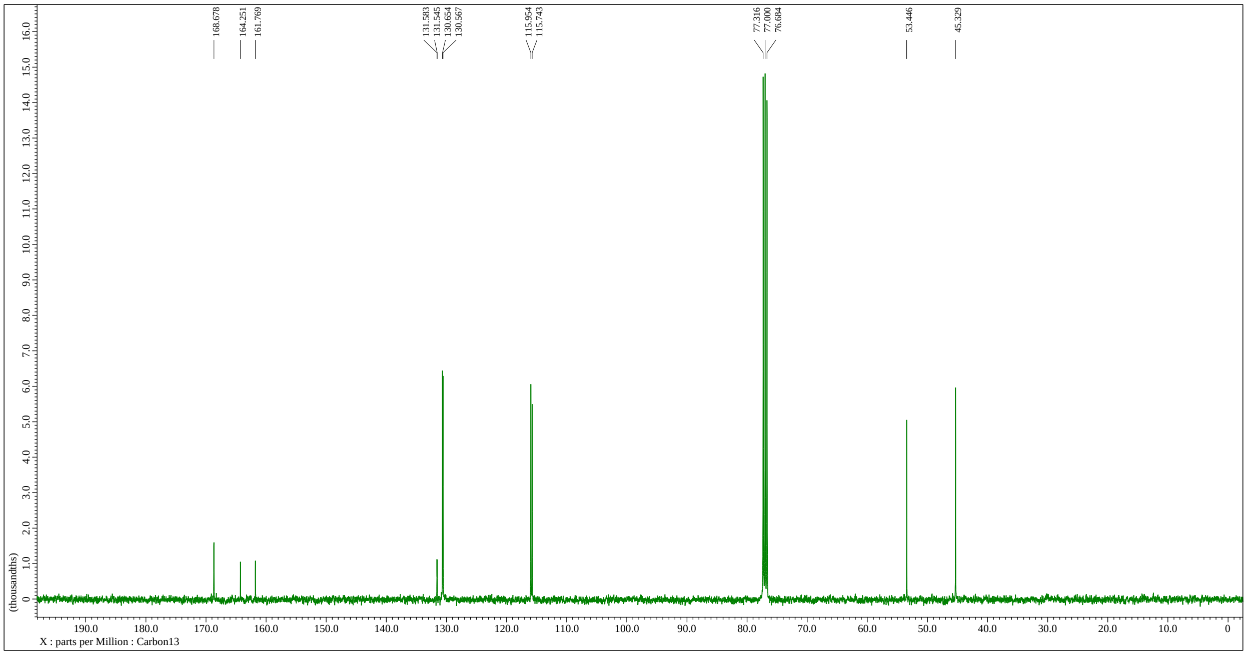

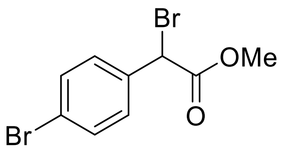

2m

^{1}H NMR

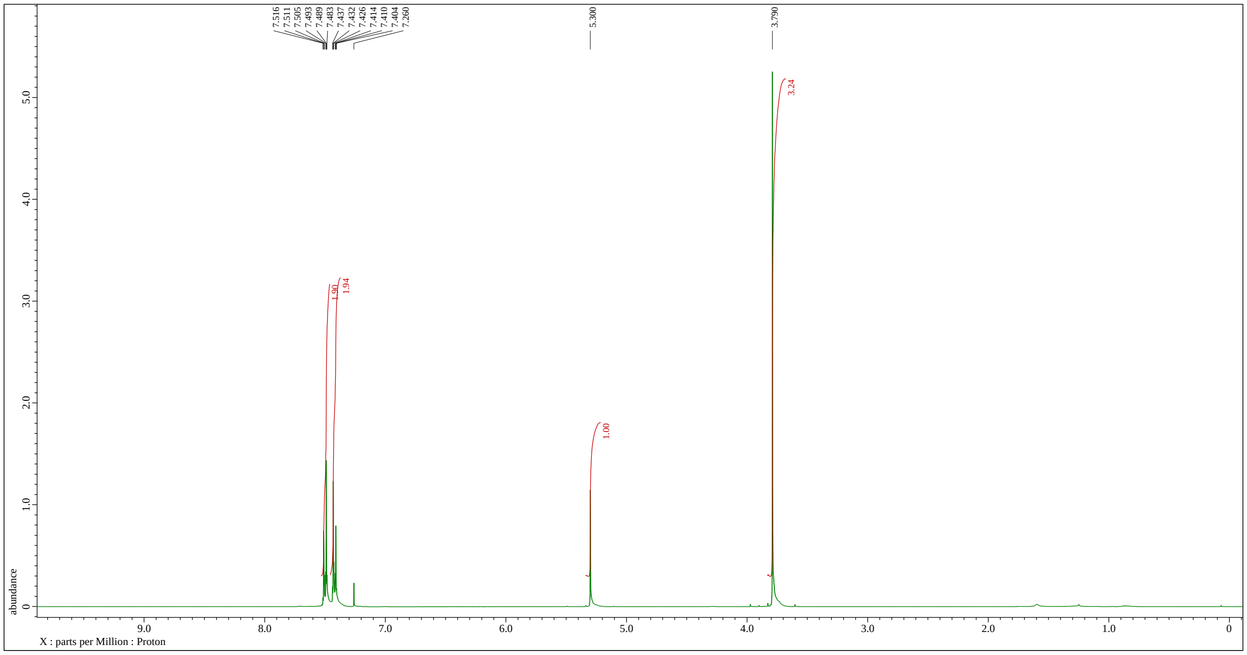

^{13}C NMR

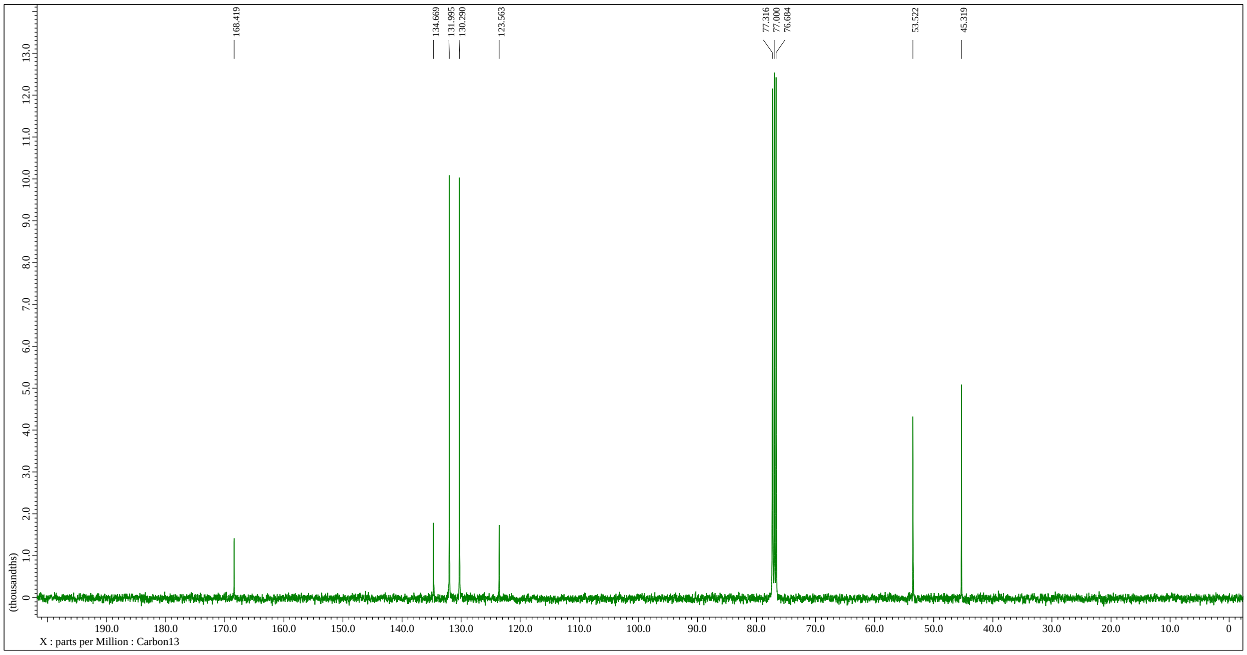

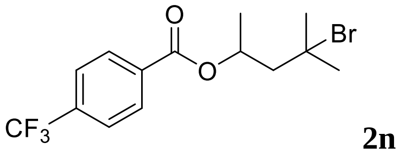

2n

^{1}H NMR

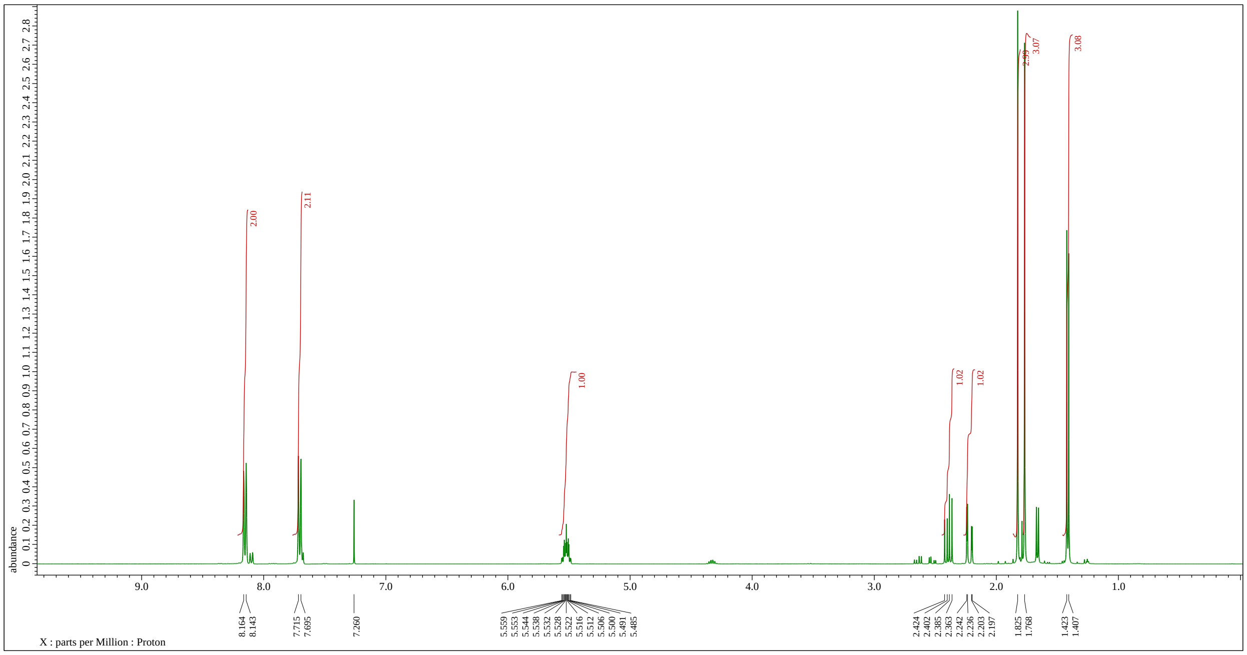

^{13}C NMR

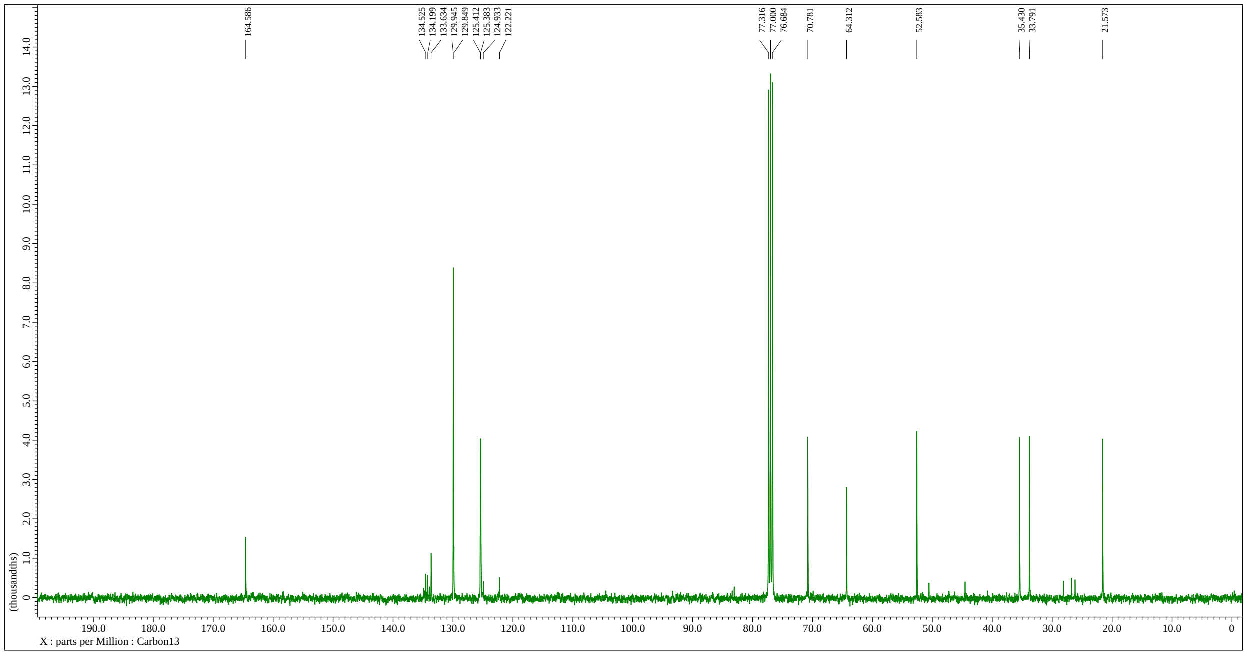

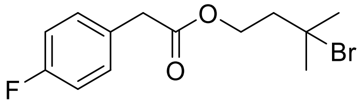

2o

^{1}H NMR

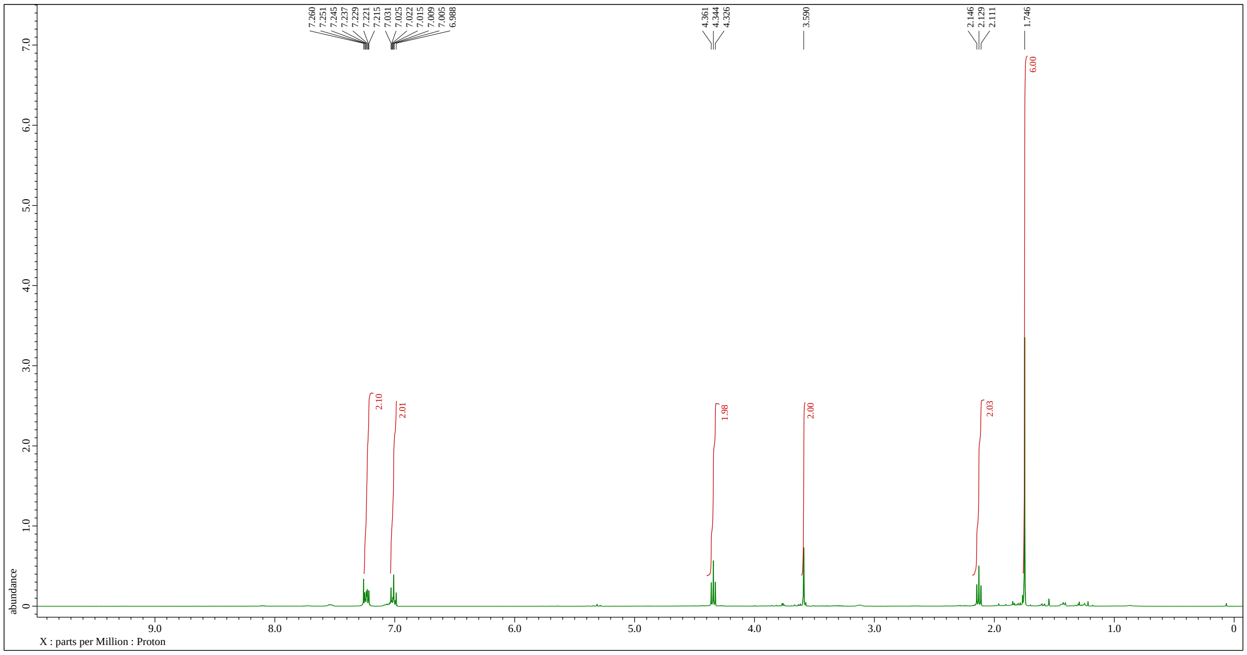

^{13}C NMR

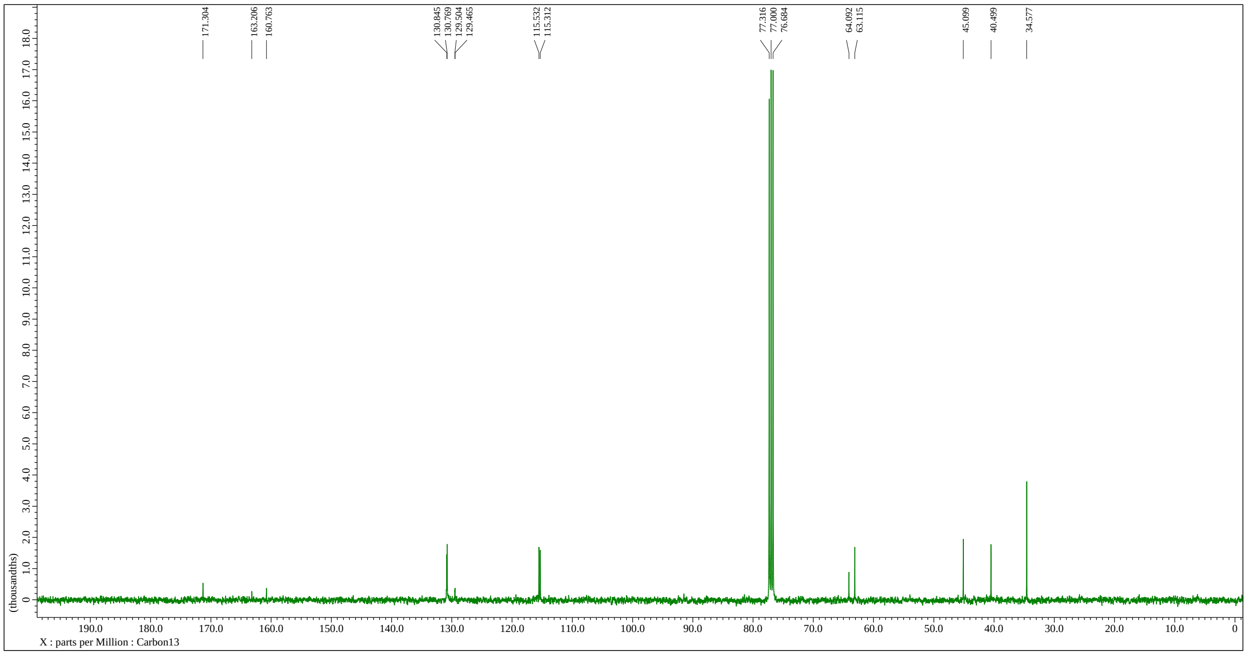

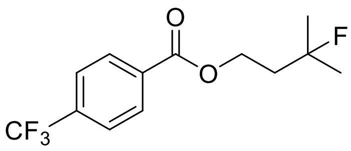

3

^{1}H NMR

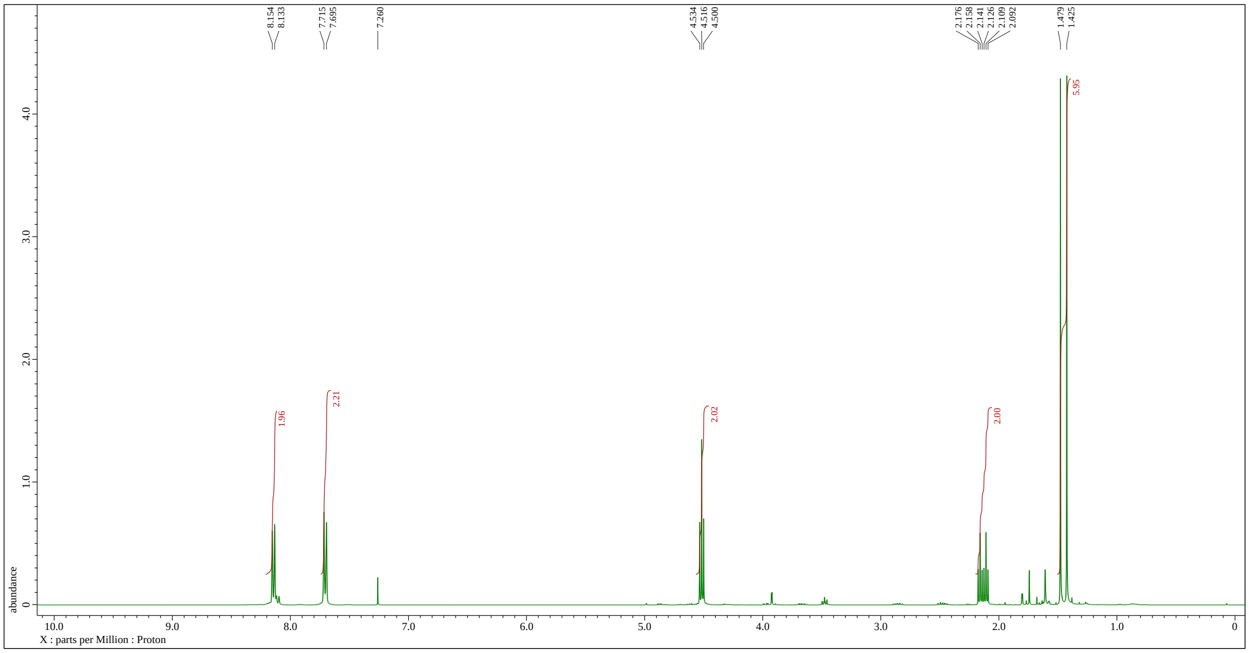

^{13}C NMR

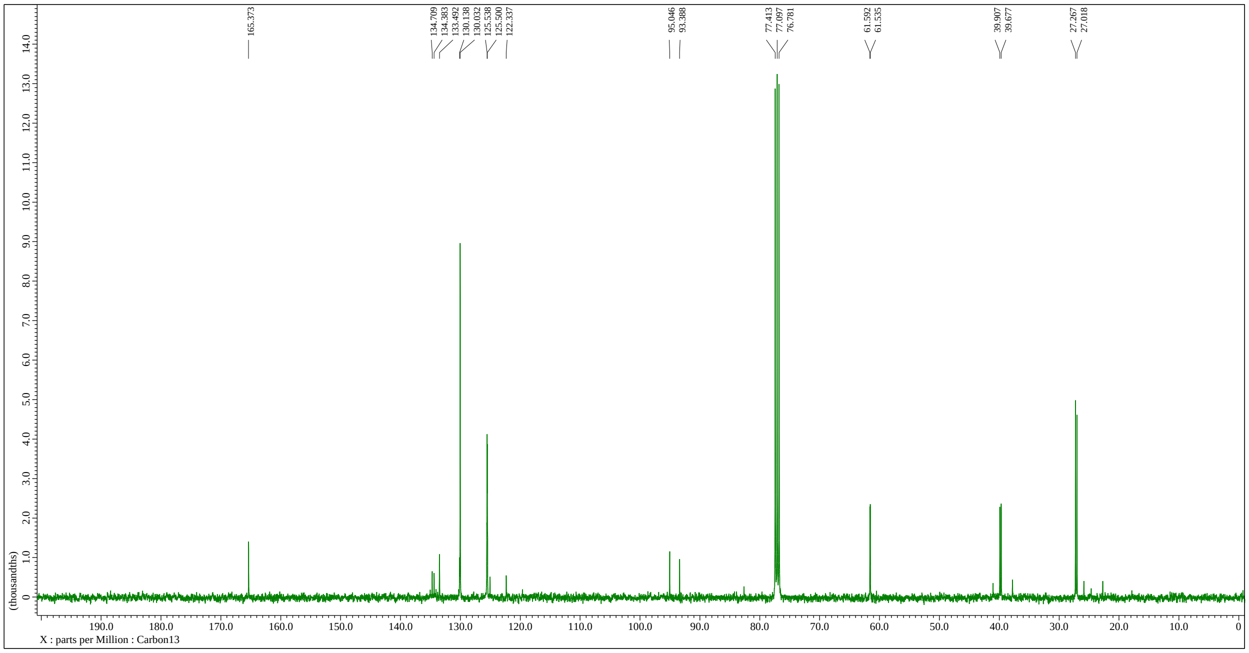

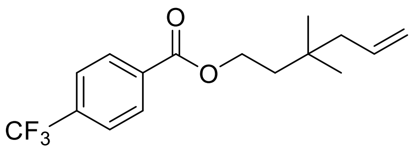

4

^{1}H NMR

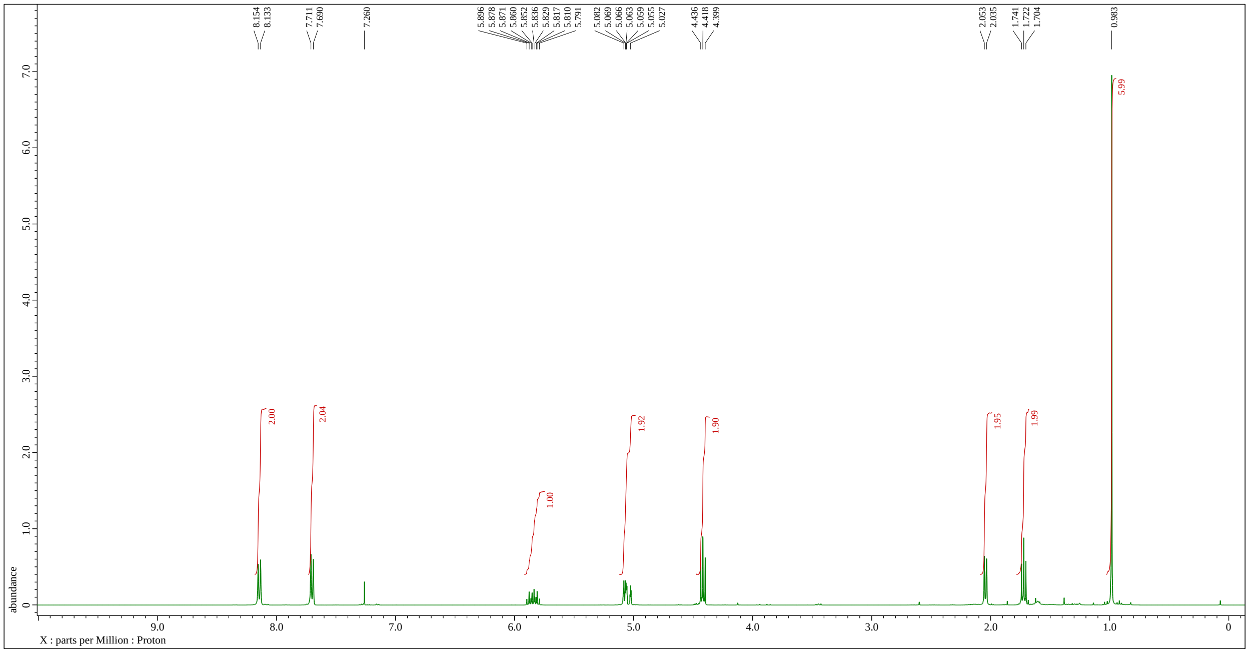

^{13}C NMR

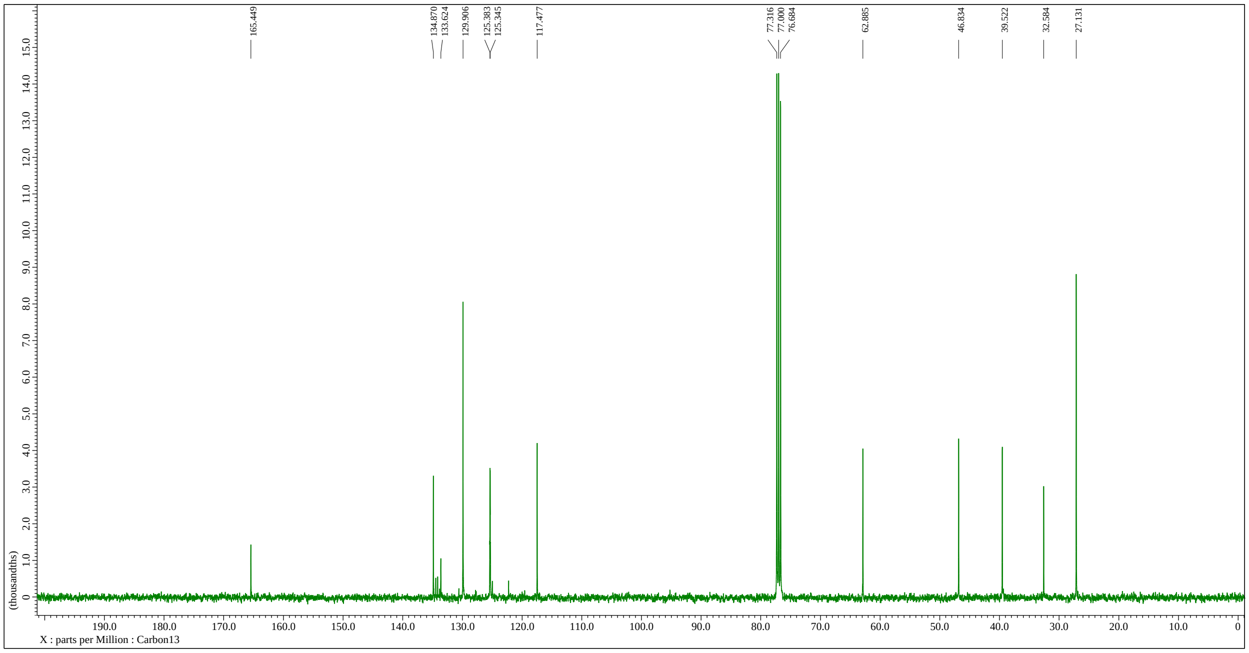

Supplement: File 1 — Experimental procedures, compound characterization data, and copies of 1H and 13C NMR spectra. [file Beilstein_J_Org_Chem-17-885-s001.pdf]
